# Models of cardiomyocyte–non‐myocyte electrical interactions

**DOI:** 10.1113/JP287295

**Published:** 2025-07-01

**Authors:** Ana Simon‐Chica, Axel Loewe, Peter Kohl

**Affiliations:** ^1^ Novel Arrhythmogenic Mechanisms Program, Centro Nacional de Investigaciones Cardiovasculares Madrid Spain; ^2^ Institute of Biomedical Engineering, Karlsruhe Institute of Technology (KIT) Karlsruhe Germany; ^3^ Institute for Experimental Cardiovascular Medicine, University Heart Center Freiburg‐Bad Krozingen, Faculty of Medicine University of Freiburg Freiburg Germany

**Keywords:** computational modelling, heterocellular coupling, non‐myocytes

## Abstract

Cardiac non‐myocytes are increasingly recognized as active contributors to cardiac electrophysiology. Fibroblasts have been shown to form connexin‐based electrotonic connections with cardiomyocytes (CM) *in situ*, and more recently, macrophages have also been found to engage in electrotonic interactions with CM. This growing evidence requires a conceptual reassessment of cardiac electrophysiology. However, studying heterocellular coupling *in situ* remains challenging. These experimental uncertainties define a scope for computational modelling and simulation. In this review, we provide an overview of computational models of heterocellular coupling across multiple spatial scales, from single‐cell interactions to whole‐organ dynamics. We start by presenting the rationale for studying cardiac heterocellular coupling that is based on clinical and experimental evidence, followed by an overview of computational modelling studies, and conclude with an outlook to future research directions.

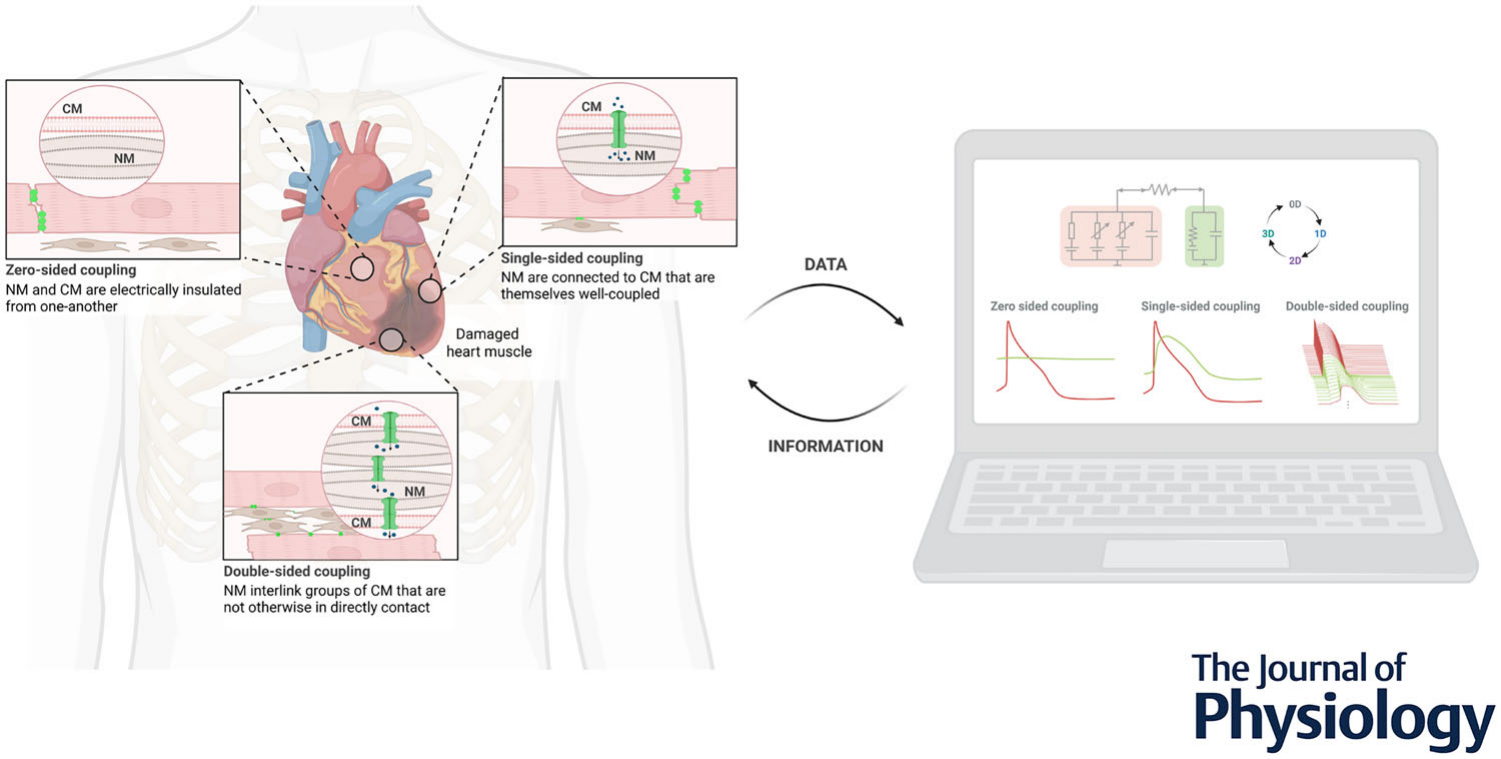

## Graphical abstract

**Abstract figure legend** Cardiomyocyte–non‐myocyte (CM–NM) electrotonic coupling. Left: conceptual contact‐based interactions between CM and other NM via connexin (Cx) proteins. Three basic scenarios are proposed: (i) ‘zero‐sided coupling’ where there are no electrical connections between CM and NM, (ii) ‘single‐sided coupling’ where NM are connected to CM that are themselves well‐coupled and (iii) ‘double‐sided coupling’ where NM interlink CM that are not otherwise in direct contact. Right: computational modelling of electrical crosstalk of CM (red) and NM (green) in the three presented scenarios.

## Introduction

The adult human heart is a complex organ, composed of billions of cells that work together, giving rise to coordinated cardiac pump activity. This coordination occurs primarily via electrical integration of cardiomyocyte (CM) activity across the heart chambers. While CM are recognized for their roles in action potential (AP) generation and propagation, cardiac non‐myocytes (NM), such as fibroblasts and immune cells, have started to emerge as contributors to cardiac electrophysiological behaviour, affecting CM resting membrane potential (RMP), excitability, AP shape, refractoriness and conduction (Simon‐Chica et al., 2023). Fibroblasts, once thought to serve merely as structural support cells and electrical insulators, have been shown to form functional connexin‐based electrotonic connections with CM *in situ* (Camelliti et al., 2004b), as evidenced by Lucifer yellow dye transfer between the heterotypic cells in healthy rabbit atria. This coupling of CM and NM can give rise to AP‐like membrane potential swings in fibroblasts of murine ventricular scar tissue (Quinn et al., 2016). More recently, macrophages (MΦ) have also been found to engage in electrotonic interactions with CM in native murine atrio‐ventricular (AV) node tissue (Hulsmans et al., 2017). Experimental evidence suggests an even wider range of indirect NM effects on cardiac electrophysiology, including neutrophils that influence ischaemia‐induced ventricular arrhythmogenesis through the generation of reactive oxygen species (Grune et al., 2022), or adipocytes that, apart from creating non‐excitable tissue regions (Sung et al., 2022), can cause electrophysiological changes in adjacent CM due to paracrine effects (De Coster et al., 2018; Lin et al., 2012), to name but a few examples.

This evidence of interstitial NM effects on CM electrical behaviour requires a conceptual reassessment of cardiac electrophysiology, challenging the long‐standing CM‐centric view, while providing new insight into the relevance of the heterocellular nature of cardiac tissue in health and disease (Lother & Kohl, 2023). However, experimental studies into CM–NM electrical coupling *in situ* remain challenging, as NM that are electrotonically coupled to CM may exhibit membrane potential dynamics that are similar to CM – so that these phenomena may easily by overlooked *in situ*. As a result, breakthrough observations of recent years have been based on novel optogenetic approaches to document cell type‐specific electrical activity, but false‐negative outcomes regarding subtle or potentially hidden effects (that manifest themselves during patho‐physiological challenges but may be absent in any given experimental setting) remain far from being avoidable in native tissue. Experimental research *in vitro* is more straightforward, but suffers from non‐physiological cell interactions, including upregulation of connexins (Cx) such as connexin 43 (Cx43), the main molecular substrate of electrical cell‐to‐cell coupling in working myocardium. This increases the potential of false‐positive outcomes in more reduced model systems.

These experimental uncertainties define a scope for computational modelling and simulation, as *in silico* approaches allow one to selectively probe individual cell behaviour in complex tissue settings, and to project experimental observations across scales, disease settings or species. Early models of heterocellular CM–NM coupling guided conceptual thinking and confirmed the biophysical plausibility of NM effects on cardiac excitation and conduction (Jacquemet, 2006; Jacquemet & Henriquez, 2008; Kohl et al., 1994; MacCannell et al., 2007). This has contributed to a growing interest in the use of computational modelling for mechanistic exploration of cardiac heterocellular coupling, and of its clinical relevance (Maleckar et al., 2009; Xie, Garfinkel, Camelliti et al., 2009).

In this review, we provide an overview of computational models of heterocellular electrical coupling across spatial scales, from single cell to whole organ. We start by presenting the rationale for studying cardiac heterocellular coupling that is based on insight from clinical and experimental models, followed by an overview of computational studies, and we conclude with an outlook to future research directions.

## Clinical and experimental evidence of cardiac heterocellular coupling

### Clinical motivation

Structural remodelling is a complex and dynamic process, present in cardiac diseases that involve alterations in cellular and extracellular components of the heart, often in response to excess functional demand, injury or disease (Kostin et al., 2002; McKay et al., 1986; Pfeffer et al., 1991; Schotten et al., 2024). These alterations affect mechanical and electrical properties of the myocardium, with potentially important clinical implications. For example, after myocardial infarction (MI), the loss of CM leads to their replacement by fibrotic scar tissue. Although cardiac scars are often perceived as inert, or even ‘dead tissue’, they are very much alive, exhibiting higher total cell density than working myocardium, with a multitude of heterocellular contributions to tissue sustenance (Rog‐Zielinska et al., 2016). While scar tissue is important for preserving ventricular integrity in the presence of dynamically changing mechanical forces, it also introduces heterogeneities in electrical excitability and conduction, and in passive mechanical properties due to excess deposition of the extracellular matrix (ECM), produced largely by activated fibroblasts (Holmes et al., 2016). These heterogeneities increase the risk of conduction blocks and re‐entry circuits, predisposing the heart to arrhythmogenesis (Cluitmans et al., 2023; Nguyen et al., 2014).

Interestingly, several clinical observations challenge the simplistic view that scars purely represent an electrical insulator. Reconnection of conduction across atrial ablation lines, for instance, has been observed in a majority of patients (including asymptomatic ones) that were electrophysiologically remapped 3 years after the original procedure (Pratola et al., 2008). This suggests the presence of trans‐scar conduction, which perhaps is one of the reasons for the fact that about a third of atrial ablations require a repeat procedure (Sanchez‐Somonte et al., 2024). While incomplete, transient, or non‐transmural lesions might explain the recurrence of electrical conduction across atrial ablation lines, this explanation would not account for conduction across post‐surgery scars, such as after transplantation, as these lesions are undoubtedly continuous and fully transmural. Nonetheless, recipient‐to‐donor heart electrical coupling has been observed in about 10% of heart transplantation recipients (Lefroy et al., 1998), occasionally requiring ablation of electrical conduction pathways across post‐transplantation scars (Rothman et al., 1995). These clinical reports underscore the plausibility of trans‐scar conduction in the heart. Whether this is based on cardiac heterocellular coupling is not known, motivating further research.

### Experimental observations

The diversity of the cellular composition of the heart has been known since the earliest anatomical assessments of myocardial structure. Quantitative studies of the multicellular composition of the heart initially relied on histology (Adler et al., 1981) and electron microscopy (Nag, 1980) of native tissue. These methods were later complemented with flow cytometry studies of isolated cardiac cells, allowing a more precise delineation of cellular identities (Pinto et al., 2016). However, the latter approach may be biased toward cell types that best survive the enzymatic digestion protocols used during sample preparation. Regardless of assessment method, and despite occupying a smaller volume fraction than CM, there is agreement now that NM account for the vast majority of cardiac cells (Ivey & Tallquist, 2016; Pinto et al., 2016; Simon‐Chica et al., 2023).

State‐of‐the‐art multi‐omic approaches have advanced our knowledge of the cellular make‐up of the heart (Lother & Kohl, 2023), identifying at least 11 major cell types and over 20 subtypes in human myocardium. Beyond CM, these include fibroblasts, immune cells (myeloid and lymphoid cells), mural (pericytes and vascular smooth muscle cells) and endothelial cells (Kanemaru et al., 2023; Litviňuková et al., 2020; Tucker et al., 2020). Other cell types such as adipocytes, neurons, Schwann cells, or melanocytes, and their roles in the patho‐/physiology of the heart, are receiving increasingly more attention (Achanta et al., 2020; Iacobellis, 2022; Koenig et al., 2022; Levin et al., 2009). Multi‐omic approaches can provide a more comprehensive view (compared with histology) into the dynamics and diversity of NM (sub‐)populations, particularly during physiological haemodynamics and pathological remodelling (Kuppe et al., 2022). The integration of multi‐omic approaches with fluorescence *in situ* hybridisation for spatial characterization of cell distribution patterns, or imaging of immunohistochemistry labelling, for example after 3D tissue clearing, hold promise for unbiased insight into the presence and distribution of cardiac NM, and into their spatial interrelation with CM in physiology and disease (Chan et al., 2024; Kanemaru et al., 2023; Palmer et al., 2024).

**Figure 1:**
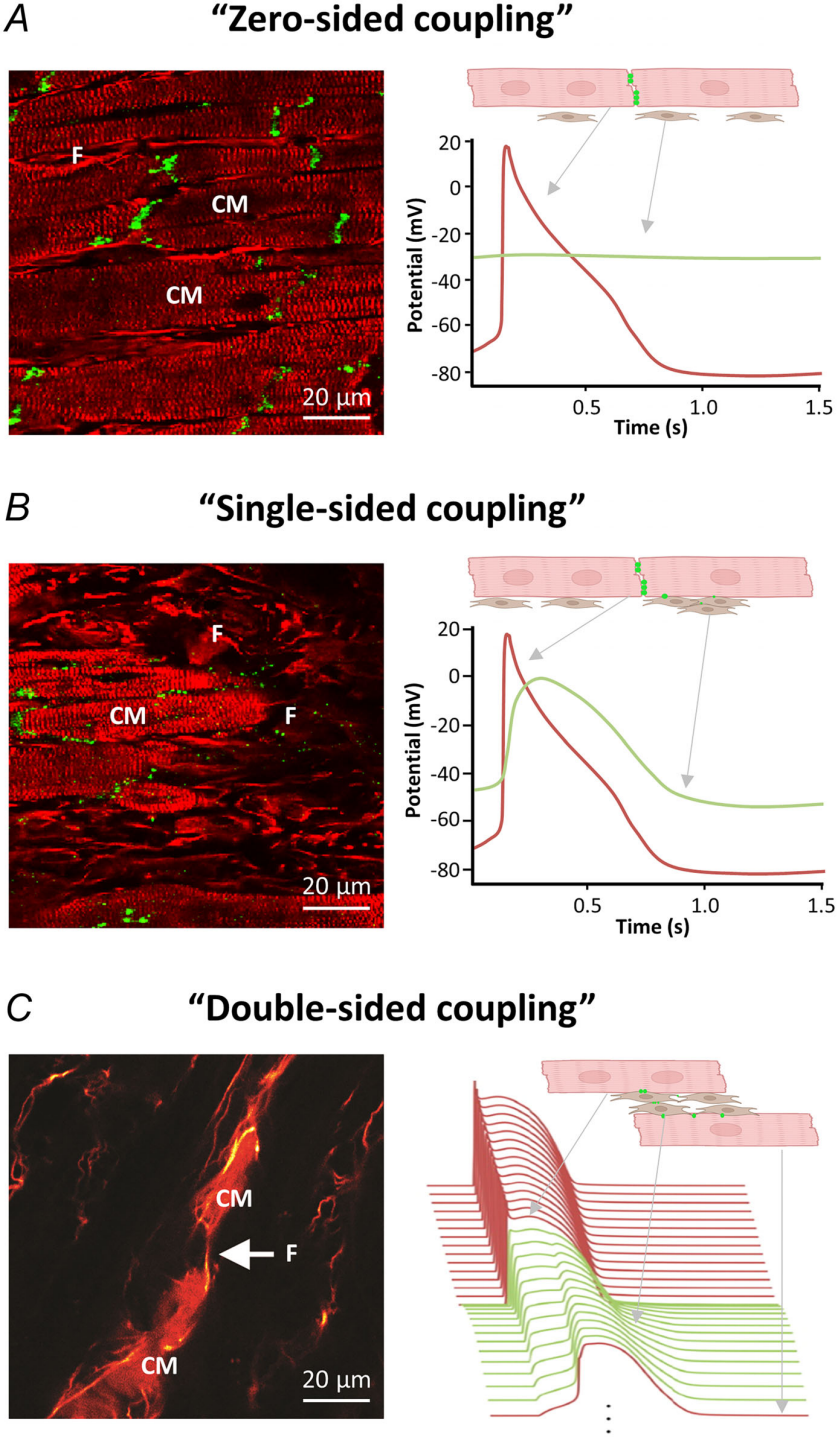
Conceptual illustration of cardiomyocyte–non‐myocyte (CM–NM) electrotonic interactions. *Left*, histological images showing connexin43 (green dots), CM (striated red) and fibroblasts (red, F) in *A* and *B*, and Lucifer yellow spread through a heterocellular chain of CM and fibroblasts in *C*. *Right*, computational modelling of electrical crosstalk of CM (red) and NM (green) in three scenarios. *A*, in ‘zero‐sided coupling’ where NM and CM are electrically insulated from one‐another, the CM action potential (AP) (red curve) is not affected by the fibroblast (green curve, showing a resting membrane potential of about ‐30 mV). *B*, in ‘single‐sided coupling’ where NM are connected to CM that are themselves well‐coupled, CM can ‘AP‐clamp’ a fibroblast. *C*, in ‘double‐sided coupling’, NM interlink groups of CM that are not otherwise in direct contact. This can support passive conduction of AP‐like signals from one group of CM (top) and trigger *de novo* generation of an AP in more distal CM. While zero‐sided coupling presumably dominates in physiological settings, single‐sided coupling may arise in reactive interstitial fibrosis (e.g. in chronic haemodynamic overload), and double‐sided connection could explain some of the *in situ* behaviour observed in replacement fibrosis (e.g. trans‐scar conduction post‐ablation, post‐surgery, post‐myocardial infarction). Sources of histology images: Camelliti (2004a, 2004b).

In the context of cardiac electrophysiology, NM (and the ECM) have traditionally been viewed as electrical insulators. This scenario can be conceptualised as ‘zero‐sided coupling’, where there is no electrical connection between CM and NM (Fig. 1*A*, Kohl & Camelliti, 2007; Kohl & Gourdie, 2014). Computational modelling (Fig. 1*A*, right) shows the CM AP (red curve) and the fibroblast membrane potential (green curve) in the absence of electrical coupling. This is, presumably, the most common scenario in native tissue, where NM serve as electrical insulator, in keeping with the observation that less than 3.5% of connexins in healthy rabbit atrial and ventricular tissue are located at heterocellular contact sites (Kohl & Camelliti, 2012).

However, two alternative modes of heterocellular electrical interaction are plausible: ‘single‐sided’ and ‘double‐sided’ (Fig. 1*B* and *C*, respectively). Single‐sided connections involve NM coupled to CM that are themselves electrically well connected: here, NM act as a passive electrotonic load on CM, with the extent of electrophysiological effects on CM (and NM) determined chiefly by the capacitance and the RMP of NM, as well as their coupling conductance to CM. Computational modelling in Fig. 1*B* shows how CM can ‘AP‐clamp’ the membrane potential of a fibroblast. Double‐sided connection refers to the scenario where NM interlink groups of CM that are not otherwise directly coupled electrically (Kohl & Camelliti, 2007, 2012). These interlinking connections are not limited to ‘one NM per CM pair’, as cardiac fibroblasts, for example, can be interconnected via homotypic gap junctions that support multicellular fibroblast–fibroblast interactions *in situ* and *in vitro*. This concept is illustrated in the computational model shown inf Fig. 1*C*, where NM can support passive conduction of AP‐like signals from one group of CM (top) and trigger *de novo* generation of an AP in more distal CM. Double‐sided coupling allows cardiac fibroblasts to form conducting bridges between CM, as shown in cell cultures (Gaudesius et al., 2003; Goshima & Tonomura, 1969; Rook et al., 1992).

Both single‐ and double‐sided coupling would be expected to be upregulated in diseases involving trans‐differentiation of fibroblasts to myofibroblasts, present in larger numbers and expressing elevated levels of Cx43 (Zhang et al., 2010). This could have clinical and therapeutic implications, for example by affecting CM excitability, or by enabling trans‐scar conduction. In this context, targeting NM to reduce the incidence of their homo‐ and heterotypic electrical connections could, for example, make cardiac ablation lines more durably insulating.

#### Key experimental findings *in situ*

First indirect evidence for CM–fibroblast capacitive and electrotonic coupling *in situ* involved the use of ‘floating’ double‐barrelled microelectrodes that were inserted into subendocardial layers of spontaneously beating atria of frog (Kohl et al., 1992) and rat hearts (Kohl et al., 1994). These studies showed that changes in the RMP of NM, presumably fibroblasts, were temporally correlated with the AP in neighbouring CM. The first definitive evidence of heterocellular cytosolic connections in native cardiac tissue was found in dye transfer studies in rabbit sinoatrial node (Fig. 1*C*, Camelliti et al., 2004b). Although Cx40 was the predominant isoform found at homotypic contact points between fibroblasts, heterocellular coupling was dominated by Cx45. Immunohistochemical experiments in sheep post‐infarct myocardium revealed an increase of Cx45 expression in fibroblasts shortly after injury, while Cx43 expression started to rise from 1 week post‐infarction (Camelliti, 2004a), highlighting the potential for temporally varying effects of cardiac heterocellular coupling in lesioned tissue.

Subsequent studies explored the electrophysiological effects of heterocellular coupling in lesioned myocardium using optical mapping of Langendorff‐perfused hearts. This, for example, revealed electrical propagation of AP‐like signals through large transmural infarcts 8 weeks after coronary occlusion in rabbit heart (Walker et al., 2007). These observations were followed up by other studies, showing cardiac excitation wave propagation, albeit attenuated, in the border zone of myocardial infarcts (Ripplinger et al., 2009; Saba et al., 2008). Despite initial scepticism, Quinn et al. definitively confirmed heterocellular electrical coupling between NM and CM *in situ* through optical mapping of transmembrane potential reporters that had been genetically targeted exclusively to NM, providing direct functional evidence of AP‐like membrane potential changes in NM in the border zone of mature ventricular cryo‐lesions in mouse hearts (Quinn et al., 2016). This was subsequently corroborated at the cellular level in a study using multiphoton laser‐scanning microscopy to measure electrical activity at the cellular level in CM and directly neighbouring NM in the border zone of post‐MI murine scars (Rubart et al., 2018). These studies highlight the importance of optical methods for exploring specific contributions of different cell types to cardiac electrophysiology (Johnston et al., 2017; Zgierski‐Johnston & Schneider‐Warme, 2021), including heterocellular electrical crosstalk.

Other NM, such as MΦ, have also been identified as contributing to cardiac electrophysiology. However, relatively few studies have specifically addressed the effects of heterocellular coupling between MΦ and CM experimentally. Hulsmans et al. (2017) showed that MΦ can be coupled, via Cx43, to CM of the AV node of healthy mice. Using optogenetic targeting of light‐activated ion channels to allow selective depolarization of MΦ, or cell type‐specific Cx43 deletion in cardiac tissue‐resident MΦ, they found that resident MΦ can alter CM electrophysiology, affecting AV conduction in isolated mouse hearts. Supporting these findings, it was later confirmed that MΦ can modulate CM electrophysiology via gap junctions in an *in vitro* model (Fei et al., 2019). This study identified KCa3.1 as a significantly upregulated ion channel protein in MΦ, following interventions mimicking MI; inhibiting this channel reduced post‐infarction arrhythmogenesis *in vivo* (Fei et al., 2019).

Most studies examining functional implications of heterocellular coupling have focused on pathological conditions. Understanding the role of CM–NM coupling in the myocardium of healthy organisms is essential to evaluate its effects within a physiological context. Based on the lack of reports highlighting overt effects of CM–NM electrical coupling in non‐injured working myocardium, and on the reported absence of detectable readouts in remote tissue even in studies that find CM–NM crosstalk in cardiac lesions (Quinn et al., 2016), we suggest that NM are unlikely to have substantial overt electrophysiological effects in the working myocardium of healthy organisms. The situation may be different in specific sub‐regions of the heart, such as the sinoatrial or AV nodes, which have a particularly high density of fibroblasts and MΦ, compared with other regions of the mammalian heart.

While it is widely recognized that cardiac electrical propagation is enabled by Cx‐based gap junctions, alternative non‐canonical mechanisms, such as capacitive (De Mazière et al., 1992) and ephaptic coupling (Mori et al., 2008) have been proposed. The two should not be confused with one another. When the capacitive current of two very closely juxtaposed membranes changes the electrical potential, interactions would be referred to as capacitive coupling. In contrast, when ion movements alter the ionic composition in the cleft space between those membranes, this may drive ephaptic interactions.

Early exploration of putative capacitive coupling between CM and fibroblasts concluded that this mode of interaction was unlikely to be of patho‐/physiological relevance, as model calculations – based on painstaking electron‐microscopic reconstructions of an unusually large area of CM–NM membrane approximation – concluded that the resulting current would have been approximately one third of that through a single gap junctional channel (De Mazière et al., 1992).

Ephaptic coupling occurs at sites where two cell surface membranes are in close proximity and form a diffusion‐restricted extracellular fluid volume – such as in the perinexus of cardiac myocytes. AP‐associated ion fluxes from a ‘proximal’ cell into this narrow, 10–20 nm‐wide extracellular space then give rise to local changes in extracellular ion concentrations that can depolarize a second ‘distal’ cell, potentially triggering an AP without a need for Cx‐based coupling (Gourdie, 2019; Hoeker et al., 2020). Indeed, experimental and modelling studies suggest that ephaptic coupling may be relevant, in particular when gap junctional coupling is diminished (Lin et al., 2022), but that the efficacy of ephaptic coupling is reduced when cleft widths exceed 30–40 nm (George et al., 2015; Ivanovic & Kucera, 2021; Veeraraghavan et al., 2018). In this context, it is important to remember that – with the exception of intercalated discs – the CM outer surface is covered (both *in vivo* and *in vitro*) by a basement membrane that is 50–70 nm thick (Langer et al., 1982). *De novo* formation of ephapses (or of Cx‐based coupling) involving CM therefore requires the other cell to break through that basement membrane (if the second cell is a CM, too, then that would have to occur at both cell surfaces). For ephaptic coupling, the two cell surfaces further need to form a diffusion‐limited cleft space. Interestingly, CM–NM contacts *in vivo* often involve localized, discrete and punctate sites where cell membranes are in close apposition over minute contact areas. These connections can be formed by tunnelling nanotubes, extending from NM towards the sarcolemma of CM (Jünger et al., 2022; Quinn et al., 2016); their role for cardiac electrophysiology is as yet unclear.

Intriguingly, a recent study reported that, even after knocking out Cx43 in channelrhodopsin‐expressing fibroblasts, optical depolarization of NM in cardiac scar tissue can still result in propagated electrical activation (Wang et al., 2023). As a note of caution, the burden of proof for cell type‐specific protein expression, and for maintenance of such cell‐type specificity during pathological remodelling, is daunting. This has been illustrated in one of the earliest studies observing ‘optical pacing’ of hearts after NM‐targeted channelrhodopsin expression: while large parts of the myocardium indeed showed cell NM‐specific targeting, painstaking analysis of large tissue volumes discovered patches of myocardium where CM also expressed the light‐activated ion channel, rendering experiments inconclusive (Johnston et al., 2017). Clearly, the roles of other connexins, of ephaptic coupling, and of tunnelling nanotubes in CM–NM crosstalk warrant further exploration.

### Key experimental findings *in vitro*

As early as the 1960s, effective synchronization of distant CM (≥150 µm), interlinked solely by multiple NM, was observed *in vitro* (Goshima, 1970; Goshima & Tonomura, 1969). Later, in 1992, it was demonstrated that fibroblasts can electrically interconnect CM via passive signal conduction, aided by the high membrane resistance and low membrane capacitance of the NM (Rook et al., 1992). Using double whole‐cell patch‐clamp experiments in co‐cultures of neonatal rat CM and fibroblasts, they showed CM‐like AP in electrotonically coupled fibroblasts. The distance over which such coupling may support propagation of excitation between rat CM *in vitro* was later assessed in a structured co‐culture system, which revealed that fibroblast inserts can passively transmit electrical activation over distances of up to 300 µm (Gaudesius et al., 2003), all in keeping with the double‐sided connection concept introduced above. Assessing effects presumably dominated by single‐sided coupling optical mapping of strands of cultured neonatal rat CM, coated with myofibroblasts (Miragoli et al., 2006), a biphasic relationship between conduction velocity (CV) and myofibroblast density was observed, suggesting that myofibroblasts affect cardiac conduction by cell density‐dependent gradual depolarization of CM. Additional studies demonstrated that reducing heterocellular gap junctional coupling by siRNA, or increasing it by overexpression of Cx43, influences cardiac electrical propagation *in vitro* (Miragoli et al., 2006; Zlochiver et al., 2008). Overall, results are reminiscent of the biphasic dependence of CV and AP upstroke velocity on the concentration of extracellular K^+^ which, by modulating the RMP, can induce the well‐known phenomena of ‘superexcitability’ (reduced distance between RMP and threshold for AP induction) and, as a consequence, ‘supernormal conduction’ (Kagiyama et al., 1982; Shaw & Rudy, 1997; Spear & Moore, 1974). Similarly, while AP conduction isochrones are crowding in a rabbit model 2 months post‐MI (indicative of slowed conduction in the peri‐infarct zone), once within the central infarct zone, CV increases (in cases even exceeding that observed in healthy myocardium; Walker et al., 2007). These, at times counter‐intuitive, findings define a need for further characterization of NM‐mediated effects on cardiac conduction, which are likely to involve elements of all three: zero‐, single‐ and double‐sided connections of NM with CM.

### Use of non‐myocytes to alter cardiac conduction

Targeted modulation of CM–NM crosstalk *in vivo* could be used to modulate structural and functional remodelling following myocardial injury (Rog‐Zielinska et al., 2016). In this context, NM genetically modified to overexpress Cx43, have been demonstrated to have anti‐arrhythmic effects. *In vivo* studies have shown that injecting NM expressing Cx43 (Roell et al., 2007), or application of Cx43 viruses to transduce cells within left ventricular scar tissue, significantly lowers the probability and duration of tachyarrhythmias induced by burst pacing in murine hearts (Mohr et al., 2026; Roell et al., 2018). This beneficial effect of increasing heterocellular crosstalk may be related to reduced electrical heterogeneity in general, or specifically to enhanced trans‐scar conduction which – if fast enough – could render lesioned tissue electrically ‘transparent’. Note that this concept is opposite to the idea of reducing heterocellular coupling in ablation lines, where the therapeutic benefit arises from local termination of electrical excitation wave propagation: the direction of targeted modification of heterocellular electrical coupling depends on the electrophysiological context and on the therapeutic purpose (recover normal conduction or stop aberrant excitation waves).

Another inspiring strategy for addressing cardiac conduction disorders involves the use of tissue‐engineered electrical conduits (Cingolani et al., 2014). Such conduits have been created using paramagnetic beads functionalized with surface‐conjugated antibodies that selectively bind to neonatal rat CM or human stromal cells. By applying a linear magnetic field, elongated heterocellular tissue strands were formed, which are structurally and functionally integrated. *In vitro* experiments demonstrated that these conduits can successfully synchronize the electrical activity of non‐connected CM regions in neonatal rat heart cultures. Subsequent *in vivo* validation involved attaching the ends of preformed CM/NM strands to the epicardium of the right atrium and right ventricle of rat hearts. This intervention resulted in formation of an AV conduction pathway that enabled sequential chamber activation when the native conduction system was inhibited using methacholine. The study thus demonstrated functional electrical coupling through heterotypic cell interactions in engineered cardiac tissue, with significant potential for cardiac resynchronization therapy (Cingolani et al., 2014). Incorporating CM, even without forming a continuous conductive pathway, offers a key advantage compared with NM‐only strands: CM can act as ‘repeater stations’ (Kohl, 2014). By reconditioning the electrotonically conducted impulse through active generation of an AP (see Fig. 1*C*), they can help to maintain excitatory signal amplitude and thus extend the distance of effective electrical propagation throughout the graft's multiple mm‐long input‐to‐output pathway (Simon‐Chica et al., 2023).

### Need for quantitative computational integration

Experimental cardiac electrophysiology has gone hand‐in‐hand with the development of computational models to reproduce the electrophysiology of CM and NM, and to assess the effects of their electrotonic crosstalk (Brown et al., 2015; Loewe et al., 2025; Niederer et al., 2019; Trayanova et al., 2024). Computational models can be used to explore and assess the plausibility of hypotheses pertaining to the causes, mechanisms, and effects of heterocellular electrical interactions. In addition, computational models allow one to selectively modify and/or observe experimentally ill‐accessible processes; to explore parameter ranges where experimental studies would be associated with very high cost in terms of time and other resources; to explore questions across different spatial and temporal scales and timeframes; to improve hypothesis‐driven experimental research; and to translate biological observations across species – providing insight that is difficult to attain in biological experiments alone (Noble, 2007). This will be detailed in the next section.

## Modelling cardiac heterocellular coupling

In this section, we will focus on computational models of heterocellular electrical coupling and their role in exploring how different cardiac cell types influence each other in homeodynamic and pathological settings. We will begin by discussing differences between passive and active NM models. Then, we will make use of the terminology introduced in Fig. 1 to characterize the nature of CM–NM electrical interactions (Kohl & Camelliti, 2007; Simon‐Chica et al., 2023), focusing on single‐sided coupling where NM may act as a current sink or source for the electrical activity of the CM, and double‐sided coupling where NM enable passive impulse conduction through bridges between otherwise non‐coupled CM. These models have been important in exploring fundamental mechanisms of heterocellular interactions, such as AP and RMP changes, propagation of cardiac excitation, and conduction block. Finally, we will introduce larger models, including 3D frameworks, that aim to reflect the intricate structure of cardiac tissue *in vivo*.

### Electrical properties of heterocellular coupling

**Figure 2:**
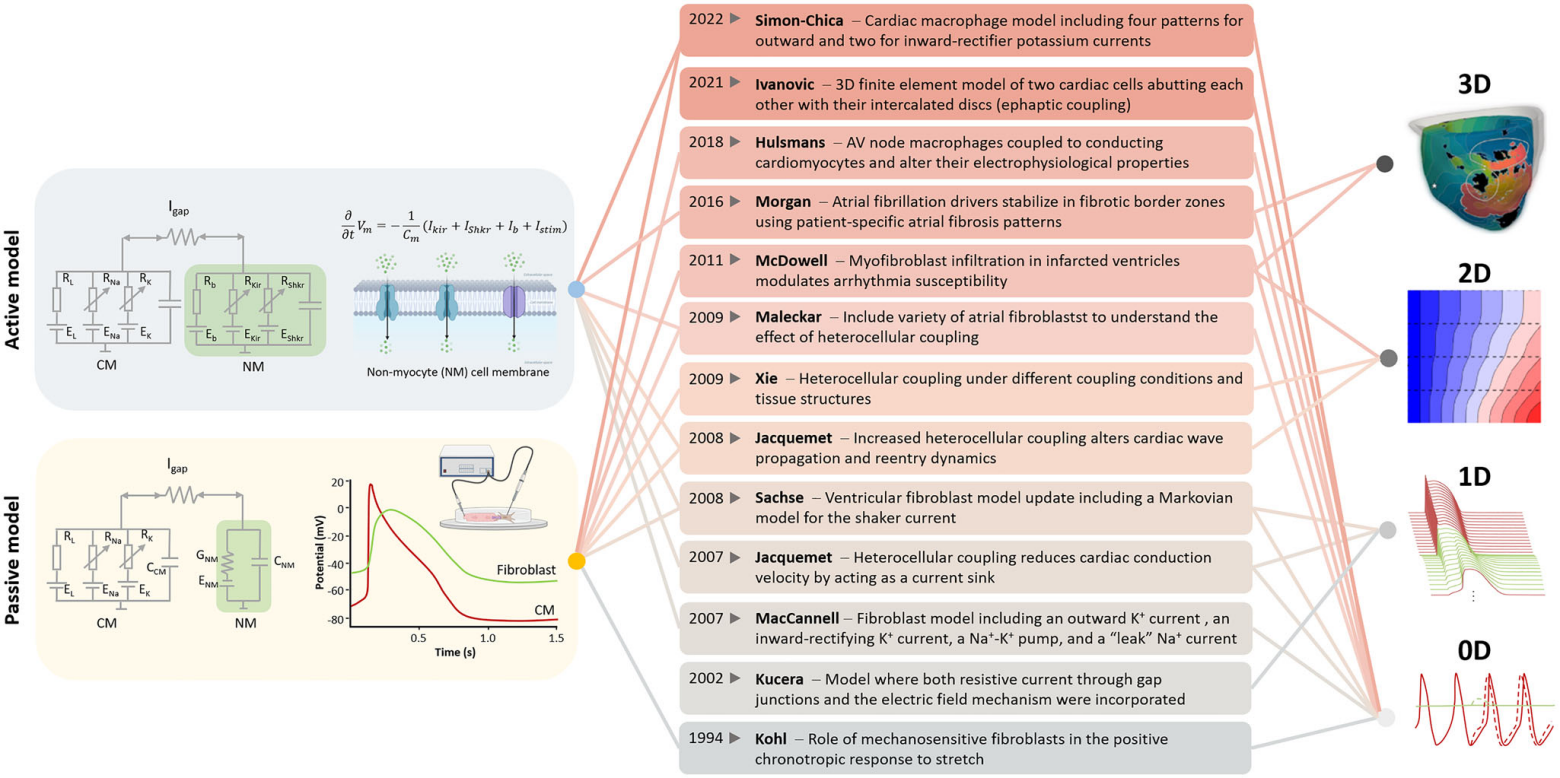
Timeline of select milestones in cardiomyocyte–non‐myocyte (CM–NM) interaction modelling. The left panels highlight two NM modelling approaches: passive (where NM are represented as a passive Ohmic resistance and capacitance) and active (illustrating that NM contain different ion channels). The middle panel provides examples of modelling studies addressing CM–NM heterocellular crosstalk. The corresponding literature references are, from bottom to top: Hulsmans et al., 2017; Ivanocic & Kucera, 2021; Jacquemet & Henriquez, 2008, 2007; Kohl et al., 1994b; Kucera et al., 2002; MacCannell et al., 2007; Maleckar et al., 2009; McDowell et al., 2011; Morgan et al., 2016; Simon‐Chica et al., 2022; Sachse et al., 2008; Xie, Garfinkel, Camelliti et al., 2009. The references included are a subset of pioneering contributions that have helped to define models which are in widespread use within the scientific community. The right panel illustrates different spatial scales addressed, from pairs of 0D (Kohl & Noble, 1996) single‐cell models and their interactions in early studies, to integrative frameworks capturing 1D (Simon‐Chica et al., 2023), 2D (modified from openCARP), and whole‐heart 3D electrophysiology (modified from (O'Hara et al., 2022)).

Mathematical models of heterocellular coupling can be split broadly into ‘passive’ and ‘active’ NM models, each defined by distinct assumptions (Fig. 2).

In the first mathematical model of cardiac heterocellular coupling (Kohl et al., 1994), fibroblasts were modelled as a passive Ohmic resistance and capacitance, electrotonically connected to CM. In this setting, the membrane potential can be represented by the ordinary differential equation

(1) $$C_{f}\;\left(\frac{dV_{f}}{dt}\right)=-G_{f}\left(V_{f}-E_{f}\right)-G_{gj}\left(V_{f}-V_{CM}\right)$$

where $C_{f}$ is the fibroblast membrane capacitance, $V_{f}$ is the membrane potential of the fibroblast, $E_{f}$ is the fibroblast's RMP, $G_{f}$ is the fibroblast membrane conductance, $G_{gj}$ represents the gap‐junctional conductance, and $V_{CM}$ is the membrane potential of the CM. This formulation has become a general representation of CM–NM coupling and, with minor variations, appears in numerous modelling studies. Although this does not represent all electrophysiological properties of fibroblasts, it allows one to independently adjust parameters such as $E_{f}$ and $G_{f}$ (Kohl & Noble, 1996; Kohl et al., 1994). Passive models were used to investigate plausible contributions of mechanosensitive fibroblasts in the sinoatrial node to cardiac responses to stretch, demonstrating that electrotonically coupled NM may increase the spontaneous pacemaker rate in a stretch‐dependent manner. Indeed, as few as 10–30 gap junctional channels (each with a conductance of 30 pS, as experimentally established in double whole‐cell patch‐clamp studies (Rook et al., 1992)) lead to electrophysiologically relevant source‐sink effects in the coupled cells. Passive models were later used also in multiscale computational models of heterocellular interactions, benefitting from their low computational demand (McDowell et al., 2013).

Following more detailed electrophysiological characterization of cardiac fibroblasts *in vitro* (Abramochkin et al., 2012; Chatelier et al., 2012; Chilton et al., 2005; El Chemaly et al., 2006; Jakob et al., 2021; Kamkin et al., 2005; Li et al., 2009; Poulet et al., 2016; Shibukawa et al., 2005), active mathematical models started to include voltage‐dependent ion currents (Jacquemet & Henriquez, 2007; MacCannell et al., 2007; Sachse et al., 2008; Sánchez, Nothstein et al., 2019, Sánchez et al., 2021), with the MacCannell model now being the most widely used. This model incorporates four fibroblast currents: a delayed‐rectifier K^+^ current, an inward‐rectifying K^+^ current, a Na^+^‐K^+^ pump and a background Na^+^ current (MacCannell et al., 2007). Magnitude and kinetics of the inwardly rectifying and delayed‐rectifier K^+^ currents are based on patch‐clamp data from freshly isolated adult rat ventricular fibroblasts (Chilton et al., 2005). The Na^+^‐K^+^ pump and the background Na^+^ currents were added to maintain K^+^ and Na^+^ ion homeostasis. The RMP of the uncoupled fibroblast is −49.6 mV and its membrane capacitance was set to 6.3 pF. This fibroblast model was coupled to ventricular CM, represented using the ten Tusscher‐Noble‐Noble‐Panfilov model (ten Tusscher et al., 2004). Fibroblast coupling significantly altered CM AP duration (APD) and morphology, resulting in partial depolarization of the RMP and APD shortening (MacCannell et al., 2007). The inclusion of time‐ and voltage‐sensitive currents in fibroblasts, activated by electrotonic depolarization when coupled to CM, led to a more pronounced shortening of the APD, when compared with passive fibroblast models. Building on this approach, an active fibroblast model was coupled to a mouse CM model (Bondarenko et al., 2004), highlighting that the fibroblast's RMP was a key determinant of the effect of coupling on CM behaviour (Jacquemet & Henriquez, 2007). Because the CM membrane potential is more negative at rest than that of fibroblasts, the latter act as a source of inward current to the CM during this phase. When the CM membrane potential is less negative than that of the fibroblast (i.e. during the AP plateau), fibroblasts act as a sink for inward current from the CM. Coupling the MacCannell fibroblast model to a canine atrial CM model (Ramirez et al., 2000), divergent effects on repolarization were observed, depending on the fibroblast RMP (Jacquemet & Henriquez, 2008): fibroblasts with a relatively negative RMP (≈−50 mV) shorten CM APD, while fibroblasts with a less negative RMP (≈−25 mV) had the opposite effect. These results can be explained by the direction of current flow during different stages of CM de‐ and repolarization. In the first case, the coupled CM are more depolarized than the fibroblast (so the latter form a current sink) during most of the AP, whereas in the second case, the late stage of CM AP is significantly prolonged because fibroblasts act as a current source, slowing full repolarization.

Further phenomenological simulations, using a detailed Markov model of time‐ and voltage‐dependent outward K^+^ currents, supported the hypothesis that the difference between CM and fibroblast RMP determines NM effects on the AP of coupled CM (Sachse et al., 2008). The Sachse model is derived from the same experimental data as the MacCannell model and thus incorporates similar ionic currents, including an inwardly rectifying current and a time‐ and voltage‐dependent outward Shaker current, though with distinct mathematical formulations. The Sachse model additionally includes a non‐specific background current to sustain a RMP of −58 mV and assumes an even smaller membrane capacitance of 4.5 pF – a critical aspect we discuss in more detail below.

Another critical factor identified in experimental and computational studies is the morphology of the CM AP itself, which can give rise to contradictory findings: heterocellular coupling shortens APD in pig CM (Nagaraju et al., 2019), whereas in mice it prolongs APD (Liu et al., 2021; Mahoney et al., 2016). The relevance of intrinsic AP dynamics for the interplay of CM with an electrophysiological system that has a reversal potential half way between CM RMP and peak AP (such as NM) has been considered in detail in the context of cation non‐selective stretch‐activated ion channels (SAC; Cooper & Kohl, 2005). The authors suggest that CM membrane potential changes are accelerated during periods where intrinsic AP dynamics move the CM membrane potential *towards* the reversal potential of SAC (e.g. shortening early APD or hastening diastolic depolarization in pacemaker cells), while the same mechanism slows CM potential dynamics during periods where the membrane potential intrinsically moves *away from* the SAC reversal potential (e.g. during late repolarization). Similar effects can be expected upon activation of non‐selective light‐activated ion channels, as long as one considers merely acute electrophysiological responses (sustained activation of ion channels may, depending on their specific ion selectivity, alter cellular ion concentrations, with knock‐on effects on CM electrophysiology). Thus, dominating net effects (e.g. on APD) of electrotonic coupling of NM to CM are, in part at least, a function of CM AP dynamics, and thus subject to differences related to functional properties (pronounced AP shape differences in atrial, ventricular, pacemaker cells), re‐/modelling (AP changes during development, ageing or disease), and species (differences in AP dynamics in murine models compared with larger mammals including human). This needs to be considered when interpreting models and experiments exploring single‐sided heterocellular connections.

Similarly, when investigating the functional relevance of double‐sided connections, CM AP properties matter. Thus, the distance over which NM may passively bridge electrical conduction of CM AP is affected by shape and duration of the input AP upstream of NM inserts. As shown by computational modelling (Simon‐Chica et al., 2023), passive fibroblast‐mediated conduction can occur over substantially longer distances when AP plateau levels are high and APD is long, indicating that murine models may underestimate the electrophysiological importance of heterocellular electrotonic coupling in larger mammals. Hyperpolarization of electrotonically coupled NM (for instance, by upregulation of NM K^+^ channels) produces a similar conduction‐curtailing effect on passive AP propagation in the model (i.e. limiting the maximum number of NM in the insert through which passive AP propagation may occur). This is consistent with experimental data where fibroblasts were transfected to express the voltage‐sensitive potassium channel Kv1.3, reducing local myocardial excitability *ex vivo* and *in vivo* (Yankelson et al., 2008).

Additional key parameters influencing passive NM‐mediated AP propagation are fibroblast capacitance and membrane resistance. For instance, minimal effects on the RMP of the ventricular CM were reported even when up to 10 fibroblasts were coupled to one CM model, resulting in a depolarization by <3 mV (MacCannell et al., 2007). This modest effect can be explained by the significantly lower membrane capacitance of fibroblasts (≈6 pF) compared with CM (185 pF), and the very high membrane resistance of fibroblasts in that model (≈6 GΩ, compared with ≈50 MΩ for CM) – using values reported for freshly isolated fibroblasts (Chilton et al., 2005; Dawson et al., 2012). This grossly underestimates fibroblast dimensions *in vivo*. Assuming a nearly spherical cell shape following enzymatic digestion, with a diameter of approximately 7–9 µm (Dawson et al., 2012), one can estimate a surface membrane area of 150–250 µm^2^ (Kohl & Gourdie, 2014). When extrapolated to the *in vivo* environment, where fibroblasts exhibit extended laminar processes and surface approximations to CM that can cover 1,500 µm^2^ or more (based on electron microscopy reconstructions (De Mazière et al., 1992) and on 3D reconstructions in optically cleared mouse hearts (Fernández et al., 2024)), their membrane capacitance would reasonably be expected to exceed freshly isolated cells by an order of magnitude, that is, reaching 60–100 pF. Similarly, membrane resistance in freshly isolated fibroblasts is significantly higher than what *in situ* measurements convey (0.5–1 GΩ; Kamkin et al., 2002; Kohl et al., 1994). This suggests that the truncated morphology of isolated cells underestimates their influence on coupled CM *in vivo*. For MΦ, if available *in vitro* data hold for the *in situ* environment, MΦ are predicted to have a membrane capacitance of 50–60 pF and a membrane resistance in the order of 720 MΩ (Simon‐Chica et al., 2022). Thus, to model representative effects of NM on cardiac tissue electrophysiology, it is important to account for structural and electrophysiologically relevant functional differences between freshly isolated NM and their *in situ* correlates.

A consistent finding across computational studies is, further, that the influence of NM on CM AP crucially depends on the degree of heterocellular gap junctional coupling, which is determined in most models by the number of heterocellular connexin channels and their conductance. This relationship is evident in studies that replicate various coupling scenarios including both single‐ and double‐sided configurations (Jacquemet & Henriquez, 2008; Seemann et al., 2017; Sridhar & Clayton, 2024). However, this dependency highlights a key limitation of computational and experimental *in vitro*/*in situ* models: the precise *in vivo* value for heterocellular coupling, and how this changes during tissue remodelling such as after myocardial injury, is unknown. Simulation studies should take this uncertainty into account and propagate it throughout the models.

Other non‐canonical forms of cell–cell crosstalk such as homo‐ and heterocellular ephaptic coupling between fibroblasts and CM have been proposed (Wang et al., 2023), as reviewed in more detail elsewhere in this themed issue (Wu et al., 2026). The question of whether or not ephaptic coupling matters for electrophysiological crosstalk *in vivo* needs further investigation, since uncertainties remain regarding the parameters used in computational modelling (such as the, perhaps unlikely, sodium channel conductance used for – non‐excitable – fibroblasts in some models (Koivumäki et al., 2014)) and the structural basis of heterocellular ephaptic coupling (e.g. the interrelation of ephaptic coupling and membrane approximation area/gap width, deliberated in section experimental observations).

While less studied than fibroblasts, cardiac MΦ have also been shown to form electrical connections with CM. As summarized in section experimental observations and reviewed elsewhere (Simon‐Chica et al., 2023), a small number of experimental studies have specifically addressed the effects of CM–MΦ heterocellular coupling on cardiac electrophysiology. Hulsmans et al. (2017) used a computational model of AV node cells (Inada et al., 2009) coupled to a passive model of MΦ (essentially using pre‐existing fibroblast representations). Their simulations support the hypothesis that MΦ coupling could modulate pacemaker activity and conduction.

A more detailed active computational model for ventricular MΦ has been developed recently, incorporating electrophysiological properties recorded from cardiac‐isolated MΦ (Simon‐Chica et al., 2022). Due to the experimentally observed diversity of cardiac MΦ electrophysiological features (Simon‐Chica et al., 2022; Simon‐Chica et al., 2024), a population of models approach was used to explore the crosstalk of variable MΦ phenotypes with CM. To construct the population of models, the conductance of four ion currents included in the model (an electronic background current, two different time and voltage‐dependent outward K^+^ currents, and the inwardly rectifying K^+^ current) and MΦ capacitance were varied systematically from 50% to 200% of their original values. Consistent with previous studies focused on fibroblasts, the most profound effects are predicted for MΦ expressing little to no inwardly rectifying K^+^ current, that is, the MΦ with the intrinsically least negative RMP.

### Cardiac conduction

An important question is how NM may affect cardiac conduction. As NM have a more depolarized RMP, compared with CM, their net effect on impulse propagation may be determined not only by representing an additional capacitance (capacitive load) but also by their depolarizing effect on resting CM (resistive load). These two effects are not simply additive because, in contrast to the theoretically expected gradual slowing of conduction with increasing membrane capacitance in simple conceptual models, increasing levels of membrane depolarization of CM by electrically coupled NM may result in a biphasic change of excitability and conduction – due to the abovementioned phenomenon of supernormal excitability and conduction (Kagiyama et al., 1982; Shaw & Rudy, 1997). More recently, using an optogenetic approach to decouple the effects of capacitive and resistive loads, it was shown that both factors contribute to conduction slowing, but the resistive effect – caused by partial depolarization of CM by NM – dominates (De Simone et al., 2020). These findings highlight that NM can modulate CV, upstroke velocity and repolarization in a complex, non‐linear and apparently beating‐rate dependent manner (Giardini et al., 2025), as a function of NM density, CM coupling, and electrophysiological properties of interacting cell types.

Motivated by these experimental findings, computational models emerged as a tool to investigate the effects of heterocellular coupling on cardiac conduction, using different approaches, from interconnected 0D cell models (Sridhar & Clayton, 2024), over 1D cable representations with NM inserts between CM (Ashihara et al., 2012; Giardini et al., 2025; Jacquemet, 2006; Sachse et al., 2008; Sánchez, Gomez et al., 2019; Zlochiver et al., 2008), and on to continuous 1D models, covered by a layer of fibroblasts (Jacquemet & Henriquez, 2007) or 2D tissue models (Ashihara et al., 2012; Jacquemet & Henriquez, 2008; Majumder et al., 2016; Morita et al., 2009; Sachse et al., 2009; Sánchez, Gomez et al., 2019; Sridhar et al., 2017; Tanaka et al., 2007; Xie, Garfinkel, Camelliti et al., 2009). By enabling *in silico* manipulation of specific cellular and tissue‐level parameters, these models provide a mechanistic framework to test hypotheses and to interpret complex interactions in ways not readily achievable through experimental approaches alone.

In a microstructure model of cardiac tissue, one‐sided coupling of fibroblasts to CM exerted non‐linear effects on RMP, CV and APD that depend on fibroblast density, fibroblast capacitance, and gap junctional coupling (Jacquemet & Henriquez, 2008). Combinations of *in vitro* optical mapping and computational modelling was used to examine the role of heterocellular coupling on CV and re‐entrant arrhythmias (Zlochiver et al., 2008). They simulated active fibroblasts (including the outward rectifying current–voltage relationship), added into a 2D layer, demonstrating that increased myofibroblast density would decrease the likelihood of re‐entry, but increase the complexity of any re‐entrant rotors once formed (increased number of phase singularities).

In spite of the rising number of experimental and modelling studies, it has remained difficult to provide a unified picture of how fibroblasts affect CM at the tissue level. A human ventricular CM model (Luo & Rudy, 1991) was coupled to a passive fibroblast model (Xie, Garfinkel, Camelliti et al., 2009) in 2D tissue models where either a layer of fibroblasts was modelled ‘on top’ of a monolayer of CM, or where fibroblasts were inserted between CM. In the first scenario (focusing on single‐sided coupling effects), increasing fibroblast density initially raised CV (due to the elevated RMP of CM, bringing them closer to threshold for sodium channel activation: superexcitability causing supernormal conduction), followed by a decrease in CV caused by the growing electrotonic load of NM on CM and the gradually progressing sodium channel inactivation. By contrast, in the model with randomly interspersed fibroblasts (as examples of double‐sided connections), they found a monotonic decrease in CV as fibroblast density increased. These findings contrast with Miragoli's experiments, which reported a biphasic relationship with interspersed fibroblast co‐cultures, whereas when fibroblasts were plated on top of the CM layer, CV was inversely related to fibroblast density. One suggested explanation of this discrepancy is that in experimental conditions where cardiac fibroblasts were plated on top of a CM monolayer, the latter may still have given rise to substantial endogenous/interspersed fibroblast content (Xie, Garfinkel, Weiss et al., 2009), which might have been high enough to obscure any transient increase in CV. In a 2D simulation study of double‐sided NM–CM coupling, longer‐range conduction up to 2.5 mm was observed for sufficiently strong gap junctional coupling and multiple myofibroblasts conducting in parallel (Sridhar & Clayton, 2025). NM–CM coupling gave rise to conduction slowing close to non‐conductive scar regions and facilitated re‐entrant activity.

More recently, another factor was identified as influencing trans‐scar conduction: the frequency of AP generation (Giardini et al., 2025). While in Langendorff‐perfused mouse hearts conduction through fibrotic tissue may be present at low pacing rates, it can fail at high stimulation frequencies, promoting re‐entrant arrhythmias. Using an *in silico* 1D model consisting of a chain of CM (Bondarenko et al., 2004) with an interspersed segment of fibroblasts (Sachse et al., 2008), stimulated at different pacing frequencies, failure of passive fibroblast‐mediated conduction occurred earlier when the pacing rate was elevated – which matched *in situ* experimental observations (Giardini et al., 2025).

More realistic 3D computational models, based on magnetic resonance imaging of rabbit heart, were used to consider the arrhythmogenic substrate resulting from heterocellular coupling in whole heart (McDowell et al., 2011). Using the Mahajan et al. model of the rabbit ventricular AP (Mahajan et al., 2008) and the MacCannell fibroblast model, NM were found to have varying effects, depending on their density. At intermediate densities, fibroblasts acted as current sinks and increased proarrhythmic APD dispersion and susceptibility to arrhythmias. However, at high densities, they acted as current sources and protected against arrhythmogenesis by inducing resting depolarization to levels that blocked electrical propagation (McDowell et al., 2011).

Other 3D computational models of human left atrial tissue used patient‐specific geometries and fibroblast distribution informed by late‐gadolinium enhancement magnetic resonance imaging data from patients with persistent atrial fibrillation (McDowell et al., 2013; Morgan et al., 2016). These models provided mechanistic insight into why ablation around fibrotic regions can be clinically effective. They showed that rotors stabilize in these areas, and when both gap junctional remodelling and fibroblast coupling are present, conduction block consistently leads to re‐entry, highlighting the critical role of NM in arrhythmogenesis. To avoid subsequent re‐entry around lesions, additional ablation of CM is required (Azzolin et al., 2023). In this context, patient‐specific computational models have been used sucessfully to predict and guide ablation targets, resulting in complete prevention of arrhythmia inducibility (Boyle et al., 2019; Trayanova et al., 2024). These models demonstrate clinical utility even without considering the potential effects of heterocellular electrical coupling. However, testing in larger cohorts of patients is necessary (Trayanova et al., 2024), and further mechanistic insights along with improvements in prediction accuracy may require patho‐physiologically realistic representation of heterocellular interactions – such as in the context of a rate‐dependency of NM effects on conduction in fibrotic myocardium. This need may be particularly relevant in ventricular models, where the thick myocardial walls create substantial transmural heterogeneity (Giardini et al., 2025). In contrast, the thinner myocardial structure in atrial tissue may contribute to a high predictive value of models, even without considering CM–NM crosstalk.

Fibrosis has generally been modelled analogously to zero‐sided connectivity, where structural changes generate interstitial conduction barriers (Bayer et al., 2016), with edge splitting (Costa et al., 2014; Gokhale et al., 2018) or percolation effects on AP conduction (Vigmond et al., 2016). In fact, most studies looked at the effects of the distribution of fibrotic tissue and only few at the effects of CM–NM coupling on cardiac electrophysiology (Sánchez & Loewe, 2022). In addition, CM in models of fibrosis may be represented as showing modified electrophysiological properties (Krueger et al., 2014), including ion channel remodelling, in part due to factors released from fibroblasts (Avila et al., 2007; Mendonca Costa et al., 2018; Ramos‐Mondragón et al., 2011), or combinations of the mechanisms mentioned above (Azzolin et al., 2023; Dasí et al., 2024; McDowell et al., 2015; Nagel et al., 2023). The choice of fibrosis modelling methodology profoundly impacts the resulting arrhythmic behaviour predictions, including rotor dynamics (Roney et al., 2016) and electrograms (Sánchez & Loewe, 2022).

In summary, understanding the nature of heterocellular interactions in the heart is essential, as it can have therapeutic implications that depend on NM cell type, electrical properties of CM and the biomedical context. In post‐MI scars, for instance, it may be beneficial to increase trans‐scar conduction (as discussed in the section *Experimental observations – use of non‐myocytes to alter cardiac conduction*), making ventricular scars electrically transparent, such as by upregulating Cx coupling and enhancing the ability of fibroblasts to act as a passive conductor or by adding electrically connected excitable cells, thereby homogenizing activity and preventing re‐entrant arrhythmias. In contrast, targeting NM could als be used to reduce the incidence of heterocellular electrical connections and prevent trans‐scar conduction after atrial ablation, for example by downregulating Cx‐coupling, which would be expected to make cardiac ablation lines more durably insulating.

## Present limitations and future directions

While substantial progress has been made in modelling CM–NM electrical interactions, several key areas remain ill‐explored. One major challenge is the development of computationally efficient multiscale models, linking subcellular processes (requiring fine temporal resolution in the order of milliseconds) to cell‐, tissue‐ and organ‐levels (capturing dynamics over seconds, minutes or longer periods, and potentially involving millions of cells), including disease‐related effects of remodelling and their potential reversal (over weeks, months or years). Most larger‐scale tissue models rely on homogenization approaches, which only consider the average effects of several dozens or hundreds of equal cells (Sánchez & Loewe, 2022). Alternative models include Kirchhoff network models, which consider individual cells but neglect their shape (Jæger & Tveito, 2023), or explicit representations of individual cells with their membrane in computational meshes, such as the extracellular‐membrane‐intracellular model (Tveito et al., 2021). Albeit coming at significant computational cost, the latter models hold the potential of mechanistically representing microscopic conduction at the level of groups of individual cells (Rosilho de Souza et al., 2024; Steyer et al., 2023) and of providing insight on how this is reflected in electrocardiograms (Loewe et al., 2025; Steyer et al., 2024). At the same time, limiting the universality and scope of models can reduce the computational cost, as reduced‐order models can provide answers to specific questions with only a few degrees of freedom very fast (Fresca et al., 2020). Scientific machine learning (Quarteroni et al., 2025) and advanced parallel computing (Sachetto Oliveira et al., 2018) also support acceleration of model throughput.

On the experimental side, key parameters such as NM cell size and coupling to other NM or CM, require *in situ* characterization, including a determination of their variability across different species, regions of the heart, and during patho‐/ physiological remodelling. Indeed, most computational studies presented here use values derived from freshly isolated cells that, at least for fibroblasts, underestimate NM size in the *in vivo* setting by about an order of magnitude (section 3.1). An additional challenge lies in the uncertainty that surrounds the gap junctional conductance values, not only for heterocellular coupling, but also for CM–CM and NM–NM interactions *in vivo* (Adams et al., 2023). Direct measurements of gap junction conductance by dual‐cell patch clamp vary widely for CM–CM (3–2,530 nS), with most studies reporting average values below 550 nS (Noma & Tsuboi, 1987; Yao, Gutstein et al., 2003; Yao, Hussain et al., 2003). This contrasts with the ≈2,000 nS typically used in computational models (Adams et al., 2023). While some of this variability may arise from experimental conditions, gap junctional conductance fundamentally depends on the number of functional gap junctional channels linking two cells, an aspect that is difficult to quantify experimentally. Systematically considering how parameter variability propagates through models by including uncertainty quantification and non‐canonical cell interaction mechanisms, such as ephaptic coupling, is important for enhancing the reliability and predictive capabilities of CM–NM interaction models (Johnstone et al., 2016). Moreover, our understanding of the 3D structure of native myocardium remains incomplete. Recent experimental work has used cleared myocardium with membrane‐bound eYFP expression in fibroblasts and MΦ to provide 3D reconstructions (Fernández et al., 2024). These high‐resolution imaging studies provide an avenue towards quantitative assessment of the abundance, dimensions, and interconnectivity of NM with one another and with CM. Initial findings reveal that fibroblasts form extensive interconnected networks, while MΦ are solitary cells in the healthy mouse heart, and that the two NM cell types cannot easily be distinguished from one another *in situ* based on morphological criteria or cell dimensions alone (Fernández et al., 2024).

In addition to structural and electrical properties, understanding the mechanical influence of CM–NM crosstalk on cardiac electrophysiology is a key aspect that needs to be further explored in experimental research and modelling. Mechanical stimuli, acting for example through SAC, may have physiological effects on the heart, including modifications in excitability, refractoriness, electrical load, or changes in heart rate and AP shape (Quinn & Kohl, 2021). Importantly, responses to stretch are not limited to CM and can be partially mediated through mechano‐sensitivity of electrically coupled NM (Kohl et al., 1994b). Although cardiac computational models continue to increase in complexity, mechano‐electric coupling is still frequently omitted, and most existing models focus predominantly on the electrophysiological and mechanical properties of CM (Buonocunto et al., 2024; Healy & McCulloch, 2005; Kuijpers et al., 2007; Niederer et al., 2006; Timmermann et al., 2017; Trayanova et al., 2004; Zabel et al., 1996). Experimental studies have shown that murine and human cardiac fibroblasts express various Ca^2^⁺‐activated transient receptor potential (TRP) channels that may convey mechano‐sensitivity (TRPC3, TRPC6, TRPM7 and TRPV4 (Peyronnet et al., 2016; Reed et al., 2014)) as well as the cation non‐selective SAC Piezo1 (Blythe et al., 2019; Emig et al., 2021). MΦ also express SAC, including Piezo1 and TRPV4, in bone marrow‐derived and tissue‐resident populations (Atcha et al., 2021; Simon‐Chica et al., 2024; Solis et al., 2019), but these have not been included in cardiac modelling as yet. In addition to mechano‐electric coupling, paracrine and autocrine signals, such as cytokines (e.g. TGF‐β, IL‐6) released during inflammation or injury, can modulate ion channel expression, location, and activity in neonatal rat CM (Kaur et al., 2013; Pedrotty et al., 2009), or downregulate cardiac Cx levels, including Cx43, in human (Lazzerini et al., 2019), while stretch (in particular in the CM‐transversal direction) in turn has been shown to upregulate Cx43 in neonatal rat CM (Camelliti et al., 2006). The temporal and spatial dynamics of these regulatory mechanisms *in vivo* remain to be explored.

Other unanswered questions relate to the old concept of supernormal conduction (Adrian, 1920; Moore et al., 1993; Spear & Moore, 1977), and the more recent suggestion that CM interspersed between NM may be functioning as repeater stations (Kohl, 2014). Experiments with Cx43 overexpression in murine ventricular lesions support the former (Roell et al., 2007), while improved trans‐scar conduction following injection of Cx43 expressing excitable cells into murine infarcts is in keeping with the latter hypothesis. Both concepts are mutually compatible, and the assessment of their presence and potential relevance in various pathological settings requires further exploration, combining experimental and computational models to better understand the biophysical mechanisms involved in trans‐scar conduction.

Developing a method for direct electrophysiological quantification of heterocellular coupling within multicellular tissue would be highly desirable. Optogenetic approaches have been used to directly assess the interplay of NM and CM electrophysiology, taking advantage of optical reporting or manipulation of the NM membrane potential (Boyle et al., 2021; Fernández et al., 2021; Funken et al., 2020; Gruber et al., 2021; Hulsmans et al., 2017; Kostecki et al., 2021; Quinn et al., 2016; Wang et al., 2023). In the context of NM‐targeted expression of light‐activated ion channels one has to consider that some of these channels can pass comparatively large currents that may not be physiological. That said, current amplitude can be titrated by light intensity (e.g. to study effects of sub‐threshold depolarization by light on cardiac electrophysiology (Langen et al., 2025)).These tools allow one to investigate the principal presence of electrotonic coupling, and to modulate electrotonic interactions of NM and CM. Additional targets include electrophysiological characterization of less studied cell types, such as lipocytes (De Coster et al., 2018), pericytes (Dalkara et al., 2025), neurones (Habecker et al., 2025), Schwann cells (Sassu et al., 2024) or endothelial cells (Pinto et al., 2016), etc., assessing functional evidence of membrane potential changes which can be used to further validate computational models (Sung et al., 2022).

In conclusion, there is a growing array of new computational and experimental tools to explore cardiac heterocellular electrical coupling *in situ*. Synergistic integration of computational tools, such as multiscale modelling, combined with experimental modalities that support cell type‐specific readouts at the whole‐organ level, from optical steering and mapping to spatially resolved multi‐omics, are poised to deepen our understanding of cardiac electrophysiology, and to guide the development of innovative, patient‐specific therapeutic strategies to combat arrhythmias more effectively.

## Additional information

### Competing interests

The authors state that they have no conflicts of interest.

### Author contributions

A.S.C., A.L. and P.K. jointly conceived and designed the content; A.S.C. drafted the text and figures; all three authors edited and revised the manuscript. All authors have read and approve the final version of this manuscript, and they agree to be accountable for all aspects of the work. All persons designated as authors qualify for authorship, and all those who qualify for authorship are designated as authors.

### Funding

The authors acknowledge support by the German Research Foundation (DFG) Collaborative Research Centre 1425 *The heterocellular nature of cardiac lesions: identities, interactions, implications* (DFG #422681845) and an individual research grant (DFG #507828355). A.L. was supported by the MICROCARD‐2 project (project ID 101172576), supported by the EuroHPC Joint Undertaking and its members (including top‐up funding by ANR, BMFTR, and Ministero dello sviluppo economico). Funded by the European Union. Views and opinions expressed are however those of the author(s) only and do not reflect those of the European Union or EuroHPC. Neither the European Union nor EuroHPC can be held responsible for them.

## Supporting information


Peer Review History
