# Supplementary material for: Models of cardiomyocyte–non‐myocyte electrical interactions
Source: J Physiol. 2025 Jul 1;604(4):1607–28. doi: 10.1113/JP287295 (PMC12909674; doi:10.1113/JP287295)
Supplement: Supplementary file 1 — Peer Review History [file TJP-604-1607-s001.pdf]

The Journal of Physiology

Computational Modelling of Cardiomyocyte-Nonmyocyte Electrical Interactions

Ana Simón-Chica, Axel Loewe, and Peter Kohl
DOI: 10.1113/JP287295

Corresponding author(s): Peter Kohl (peter.kohl@uniklinik-freiburg.de)

The referees have opted to remain anonymous.

Review Timeline:

Submission Date:	30-Jan-2025
Editorial Decision:	17-Mar-2025
Revision Received:	21-May-2025
Accepted:	04-Jun-2025

Senior Editor: Bjorn Knollmann

Reviewing Editor: T Alexander Quinn

Transaction Report:

(Note: With the exception of the correction of typographical or spelling errors that could be a source of ambiguity, letters and reports are not edited. Depending on transfer agreements, referee reports obtained elsewhere may or may not be included in this compilation. Referee reports are anonymous unless the Referee chooses to sign their reports.)

1st Editorial Decision 17-Mar-2025

Dear Dr Kohl,

Re: JP-TR-2025-287295 "Computational Modelling of Cardiomyocyte-Nonmyocyte Electrical Interactions" by Ana Simón-Chica, Axel Loewe, and Peter Kohl

Thank you for submitting your manuscript to The Journal of Physiology. It has been assessed by a Reviewing Editor and by 2 expert referees and we are pleased to tell you that it is potentially acceptable for publication following satisfactory major revision.

The referee reports are copied at the end of this email.
Please address all the points raised and incorporate all requested revisions or explain in your Response to Referees why a change has not been made. We hope you will find the comments helpful and that you will be able to return your revised manuscript within 9 months. If your article is for a Special Issue, please note that we require your revised version within 2 months (rather than 9 months) in order to keep the Special issue on track. If you require longer than this, please contact journal staff: jp@physoc.org. Please note that this letter does not constitute a guarantee for acceptance of your revised manuscript.

ABSTRACT FIGURES: Authors are expected to use The Journal's premium BioRender account to create/redraw their Abstract Figures. Information on how to access this account is here:
https://physoc.onlinelibrary.wiley.com/journal/14697793/biorender-access.
This will enable Authors to create and download high-resolution figures. If authors have used the free BioRender service, they can use the instructions provided in the link above to download a high-resolution version suitable for publication.
The link provided should only be used for the purposes of this submission. Authors will be charged for figures created on this account if they are not related to this manuscript submission.

Your revised manuscript should be submitted online using the link in your Author Tasks: Link Not Available. This link is accessible via your account as Corresponding Author; it is not available to your co-authors. If this presents a problem, please contact journal staff (jp@physoc.org). Image files from the previous version are retained on the system. Please ensure you replace or remove any files that are being revised.
If you do not wish to submit a revised version of your manuscript, you must inform our journal staff (jp@physoc.org) or reply to this email to request withdrawal. Please note that a manuscript must be formally withdrawn from the peer review process at one journal before it may be submitted to another journal.

REVISION CHECKLIST:
Check that your Methods section conforms to journal policy: https://jp.msubmit.net/cgi-bin/main.plex?form_type=display_requirements#methods.

Check that data presented conforms to the statistics policy: https://jp.msubmit.net/cgi-bin/main.plex?form_type=display_requirements#statistics.

Upload a full Response to Referees file. To create your 'Response to Referees': copy all the reports, including any comments from the Senior and Reviewing Editors, into a Microsoft Word, or similar, file and respond to each point, using font or background colour to distinguish comments and responses and upload as the required file type.

Please upload two versions of your manuscript text: one with all relevant changes highlighted and one clean version with no changes tracked. The manuscript file should include all tables and figure legends, but each figure/graph should be uploaded as separate, high-resolution files.

You may also upload:
- 'Potential Cover Art' for consideration as the issue's cover image
- Appropriate Supporting Information (Video, audio or data set: see https://jp.msubmit.net/cgi-bin/main.plex?form_type=display_requirements#supp).

IMPORTANT POINTS TO NOTE WHEN REVISING YOUR MANUSCRIPT:
LANGUAGE EDITING AND SUPPORT FOR PUBLICATION: If you would like help with English language editing, or other article preparation support, Wiley Editing Services offers expert help, including English Language Editing, as well as translation, manuscript formatting, and figure formatting at www.wileyauthors.com/eeo/preparation. You can also find resources for Preparing Your Article for general guidance about writing and preparing your manuscript at www.wileyauthors.com/eeo/prepresources.

TRANSPARENT PEER REVIEW POLICY: To improve the transparency of its peer review process, The Journal of Physiology publishes online as supporting information the peer review history of all articles accepted for publication. Readers will have access to decision letters, including Editors' comments and referee reports, for each version of the manuscript, as well as any author responses to peer review comments. Referees can decide whether or not they wish to be named on the peer review history document.

We look forward to receiving your revised submission.
If you have any queries, please reply to this email and we will be pleased to advise.

Yours sincerely,

Bjorn Knollmann
Senior Editor
The Journal of Physiology

REQUIRED ITEMS

- Please include an Abstract Figure file, as well as the Figure Legend text within the main article file. The Abstract Figure is a piece of artwork designed to give readers an immediate understanding of the Review Article and should summarise the main conclusions. If possible, the image should be easily 'readable' from left to right or top to bottom. It should show the physiological relevance of the Review so readers can assess the importance and content of the article. Abstract Figures should not merely recapitulate other figures in the Review. Please try to keep the diagram as simple as possible and without superfluous information that may distract from the main conclusion of the Review. Abstract Figures must be provided by authors no later than the revised manuscript stage and should be uploaded as a separate file during online submission labelled as File Type 'Abstract Figure'. Please ensure that you include the figure legend in the main article file. All Abstract Figures will be sent to a professional illustrator for redrawing and you may be asked to approve the redrawn figure before your paper is accepted.

- Your MS must include a complete "Additional information section" with the following 4 headings and content:

Competing Interests: A statement regarding competing interests. If there are no competing interests, a statement to this effect must be included. All authors should disclose any conflict of interest in accordance with journal policy.

Author contributions: Each author should take responsibility for a particular section of the study and have contributed to writing the paper. Acquisition of funding, administrative support or the collection of data alone does not justify authorship; these contributions to the study should be listed in the Acknowledgements. Additional information such as 'X and Y have contributed equally to this work' may be added as a footnote on the title page.

It must be stated that all authors approved the final version of the manuscript and that all persons designated as authors qualify for authorship, and all those who qualify for authorship are listed.

Funding: Authors must indicate all sources of funding, including grant numbers. If authors have not received funding, this must be stated.

It is the responsibility of authors funded by RCUK to adhere to their policy regarding funding sources and underlying research material. The policy requires funding information to be included within the acknowledgement section of a paper. Guidance on how to acknowledge funding information is provided by the Research Information Network. The policy also requires all research papers, if applicable, to include a statement on how any underlying research materials, such as data, samples or models, can be accessed. However, the policy does not require that the data must be made open. If there are considered to be good or compelling reasons to protect access to the data, for example commercial confidentiality or legitimate sensitivities around data derived from potentially identifiable human participants, these should be included in the statement.

Acknowledgements: Acknowledgements should be the minimum consistent with courtesy. The wording of acknowledgements of scientific assistance or advice must have been seen and approved by the persons concerned. This section should not include details of funding.

- Please upload separate high quality figure files via the submission form.

- Author profile(s) must be uploaded via the submission form. Authors should submit a short biography (no more than 100 words for one author or 150 words in total for two authors) and a portrait photograph of the two leading authors on the

paper. These should be uploaded and clearly labelled together in a Word document with the revised version of the manuscript. Any standard image format for the photograph is acceptable, but the resolution should be at least 300 DPI and preferably more. A group photograph of all authors is also acceptable, providing the biography for the whole group does not exceed 150 words.

- Please include a full title page as part of your main article (Word) file, which should contain the following: title, authors, affiliations, corresponding author name and contact details, keywords, and running title.

- Please ensure that the Article File you upload is a Word file.

EDITOR COMMENTS

Your manuscript has been reviewed by two experts in the field, who felt it is timely and has potential to make a valuable contribution to the field. They have, however, made several important suggestions that need to be addressed through and extensive revision to strengthen the manuscript and enhance its impact, including highlighting novel insights, and clarifying mechanisms and their physiological implications. Please update the manuscript accordingly, including a point-by-point response to the reviewers' suggestions.

Senior Editor:

For the review to be acceptable, extensive rewriting as suggested by both expert reviewers would be required to address inaccuracies and make the piece more accessible to the general reader.

REFEREE COMMENTS

Referee #1:

This solicited review by Chica et al is timely owing to several recent high impact papers demonstrating the role of non-myocyte-myocyte coupling in vivo, including the role of macrophages and fibroblasts in scar tissue regulating cardiac muscle excitability. Although the review discusses in some detail the literature regarding heterocellular coupling, it really fails to discuss the findings in a physiological context. Complex issues such as ephaptic coupling have only a cursory mention and the review mainly focuses on discussing the authors' previous contributions. While no one is denying that the authors have made substantial contributions to this field, the review fails to rise on this occasion. It is rather a plebeian summary of old reviews in this field. I would strongly recommend the following revisions to make this review non-trivial:

1)The authors talk about various mechanisms of heterocellular coupling including connexins, ephaptic signaling, nanotubes etc but a discussion should be made of how these would electrically differ in vitro and most importantly in vivo in terms of functional outcomes. I understand that modeling data may not be able for all of them but still a discussion should be made.

2) A lot of the background on experimental observations can be shortened. It is just a rehash of old papers and no insights are obtained.

3)The discussion about Connexins/Cx43 are confusing. Cx43 couples fibroblasts and myocytes and this could be arrhythmogenic but increased Cx43 could have beneficial effects on the scar tissue in terms of myocardial conduction velocity. These sections should be explained

4)I think a section should be devoted to the physiological context of heterocellular coupling

a) What are the triggers in vivo that could change the RMP of non-myocytes (stretch, ROS??). Does Change of RMP expected to occur slowly or rapidly in these instances and the single sided and double sided coupling model should be discussed in these contexts to reflect on myocyte depolarization?

b) The above discussion has also implications for recent high profile papers (Wang et al. Science, 1480-87 and Hulsman et al (Cell, 510-522). How physiological is the large current being generated in vivo in non-myocytes using these optogenetic platforms?

c) What are boundary conditions for sustained arrhythmogenesis to occur via myocyte-non myocyte coupling? What are the kinds of arrhythmias that can be generated potentially via myocyte-non-myocyte coupling? What would be the initiating events in these instances?

d) Ephaptic coupling in disease conditions is thought to have more relevance in cardiac pathophysiology, perhaps the role of myocyte-non-myocyte and ephaptic coupling in healthy hearts versus diseased hearts should be discussed.

e) Coupling properties with the cell type: myofibroblasts versus fibroblasts versus macrophage (how would the coupling outcomes differ)

5) Fig 2 should be removed. It serves no purpose rather than advertising some papers. Better to put in some conceptual figures for the reader on myocyte-non myocyte coupling discussing any of the physiological issues discussed above or in addition.

6) Transcar conduction is an interesting concept and may be clinically very important and this should be discussed in context of single sided and double sided coupling models raised by the author which I think is a good way of describing it.

7)Source sink relationships and how it affects outcomes of myocyte-non myocyte coupling should be discussed especially with regard to the diseased heart.

Referee #2:

This manuscript provides a valuable overview of current studies on heterocellular coupling in the heart, focusing on the electrical interactions between cardiomyocytes and non-myocytes (fibroblasts and macrophages) in both experimental and computational settings. It outlines the limitations of existing computational models and offers insightful perspectives on the future development of this field. The review effectively bridges clinical relevance, experimental evidence, and computational modelling, highlighting the importance of heterocellular coupling in cardiac electrophysiology. The following points require consideration by the authors.

Title:

The manuscript integrates both experimental and computational studies, with each contributing nearly 50%. The current title specifically highlights the computational modelling and may not fully reflect this balance. Consider revising it to better align with the content of the review.

Introduction:

• Conceptual Re-assessment (Line 42): Elaborate on what the "conceptual re-assessment" entails regarding cardiac

electrophysiology. Provide a clearer statement about why the existing view needs to be revised in light of the evidence of non-myocyte effects.

Clinical and Experimental Evidence of Cardiac Heterocellular Coupling:

• Clinical Evidence (Lines 71-73): Replace the reference to the review paper with citations to original research articles that provide experimental evidence of structural remodeling in cardiac diseases.

• Section Title (Lines 93-94): Since the last sentence in Lines 93-94 states that it is unknown if the clinical findings are based on heterocellular coupling, the section title may need to change as there is no direct clinical evidence.

Figure 1 Legend:

• Change "I" to lowercase "i" in "(green dots in I and ii)".

• Justify why zero-sided coupling may dominate in healthy hearts (provide evidence or reasoning). Is there a citation?

• Replace the last "while" with "and".

Experimental Observations

• Cx45 Expression (Line 171): Specify whether Cx45 expression increases or decreases.

• Direct evidence of coupling (Lines 188-180): "...demonstrating the presence of heterocellular coupling in the heart." Is direct electrotonic coupling shown or does the data show evidence of electrical interactions?

• KCa3.1 (Line 199): Correct "gene" to "channel protein." KCNN4 is the gene name.

• Capacitative Coupling (Lines 202-204): The authors should clarify their definition of capacitative coupling and how it relates to emerging concepts of ephaptic coupling in the heart. Both involve electrical field effects, ephaptic coupling, particularly in structures like the perinexal ephapse at gap junction edges, encompasses electrophysiological interactions that resemble capacitative coupling in some respects. Could the concepts introduced here as independent be the same thing? An acknowledgment of this unifying possibility as embodied by the perinexus would be useful.

• Relevance of Capacitative Coupling (Line 205): Explain briefly why capacitative coupling was deemed unlikely to be of pathophysiological relevance (cite the study's reasoning).

• Ephaptic Coupling (Lines 209-212): Acknowledge the uncertainty surrounding ephaptic coupling between different cell types. Rephrase the statement to treat the existence of heterocellular ephaptic coupling as a research question.

• Punctate Connections: Clarify what "punctate connections" refers to. Does it refer to gap junction plaques or other types of connections?

• Co-cultures: In Line 227, please use co-cultures instead of cultures.

• Line 266 "Asses" - :) maybe assess is the word you should settle on ?

• Resting and Action Potential Properties

Subtitle: Consider changing the subtitle to something like "Electrical Properties of Heterocellular Coupling" to better reflect the section's content. Action potential associated with heterocellular coupling is reflected by dynamics of ionic current.

• Equation (Line 292): Clarify if the equation given is different from the one in the appendix of the cited paper (Kohl et al., 1994) or provide the correct citation.

• NM vs. Fibroblasts: As suggested, use "fibroblasts" instead of "NM" when discussing computational models involving fibroblasts for clarity. When the NM-CM computational model was discussed, the NM seem to refer to fibroblasts. By contrast, MΦ was used when a MΦ-CM computational model is discussed. I would suggest using fibroblasts instead of NM in the computational models involving fibroblasts.

Cardiac Conduction

• Experimental Findings: Suggest moving the first paragraph in this section, which discusses experimental effects of NM on cardiac conduction, to the section on Experimental Observations for better organization. The first paragraph in this section basically discussed effects of NMs on cardiac conduction in experimental conditions. Since this section primarily focuses on computational models, this paragraph may be more suitable to be placed somewhere in the section of Experimental observations.

• "Quantitatively" (Lines 265 and 444): Consider replacing the term "quantitatively" to better emphasize the significance of computational models compared with experimental models. For example, a computational model can be used to deeply investigate the complex mechanisms that the experimental model cannot achieve - "quantitatively" may not fully convey this important nuance.

Figure 2:

• Improve the image resolution.

• Ensure the arrows in the circuit diagram for the active model are bi-directional to indicate current flow through gap junctions.

• Clarify whether the modeling circuits are also suitable for MΦ-CM interaction. If not, consider adding a circuit specifically for MΦ-CM interaction.

• The middle panel in Figure 2: It should be Hulsmans, not Hulsman. Also, I don't think Hulsmans, et al. provided a 3D computational model in that paper. Please clarify if I am wrong.

• The authors provide thoughtful perspectives regarding future directions for computational modeling of heterocellular coupling. As mentioned earlier, existence of ephaptic coupling between myocytes and nonmyocytes remain unclear, the future computational modelling may thus also consider incorporating ephaptic coupling. There are useful recent mathematical models incorporating nanoscale structures of myocyte-myocyte ephaptic coupling that could also be of service in translation to non-canonical heterocellular contacts (PMID: 34533834, PMID: 34264306).

• Authors may also consider to consistently use the first or last author for the references to avoid confusion. For example, I could not find Trayanova et al, 2011 in the citations. Maybe this citation is to the paper of McDowell, et al, 2011 in Line 811.

Present Limitations and Future Directions

• Computational Efficiency: While the review mentions the need for computationally efficient multiscale models, it could elaborate on specific techniques or approaches that could be used to achieve this goal (e.g., model order reduction, parallel computing, etc.).

• Ephaptic Coupling: As suggested, future computational modelling may consider incorporating ephaptic coupling.

Additional Points for Consideration

• Gap Junction Conductance Variability: The review mentions that the precise in vivo value for heterocellular coupling is unknown. Interesting there is equal uncertainty on measurements of GJ conductance levels between myocytes. This was discussed in a recent paper from Poelzing's group (PMID: 37681314), which when surveying historical reports concluded that conductance levels used in many models may be over-estimated by as much as an order of magnitude. Discuss the implications of this uncertainty for model predictions and suggest ways to address this issue (e.g., sensitivity analysis, uncertainty quantification).

• Mechano-Electrical Feedback: The review could also discuss the potential role of mechano-electrical feedback in heterocellular coupling. How do mechanical forces affect the electrical properties of cardiomyocytes and non-myocytes, and how can this be modeled?

END OF COMMENTS

EDITOR COMMENTS Senior Editor (Bjorn Knollmann):

Your manuscript has been reviewed by two experts in the field, who felt it is timely and has potential to make a valuable contribution to the field. They have, however, made several important suggestions that need to be addressed through and extensive revision to strengthen the manuscript and enhance its impact, including highlighting novel insights, and clarifying mechanisms and their physiological implications. Please update the manuscript accordingly, including a point-by-point response to the reviewers' suggestions.

For the review to be acceptable, extensive rewriting as suggested by both expert reviewers would be required to address inaccuracies and make the piece more accessible to the general reader.

We appreciate the feedback and suggestions provided. We have extensively rewritten the review and include a point-by-point response to the reviewers' suggestions for your consideration.

REFEREE COMMENTS

Referee #1:

This solicited review by Chica et al is timely owing to several recent high impact papers demonstrating the role of non-myocyte-myocyte coupling in vivo, including the role of macrophages and fibroblasts in scar tissue regulating cardiac muscle excitability. Although the review discusses in some detail the literature regarding heterocellular coupling, it really fails to discuss the findings in a physiological context. Complex issues such as ephaptic coupling have only a cursory mention and the review mainly focuses on discussing the authors' previous contributions. While no one is denying that the authors have made substantial contributions to this field, the review fails to rise on this occasion. It is rather a plebeian summary of old reviews in this field. I would strongly recommend the following revisions to make this review non-trivial:

We understand the concern regarding the need to place the discussion of heterocellular coupling in a physiological context. Although there is hardly any published data on NM-CM coupling effects on cardiac electrophysiology in healthy myocardium (in our experience at least in part because of the challenges associated with publishing ‘negative’ data), we have now included a discussion on the topic. We also recognize that ephaptic coupling was mentioned too briefly in the original manuscript. In response, we have slightly expanded our discussion of this topic and additionally we refer to another paper in the same Special Issue of JP dedicated to ephaptic coupling. We have expanded the number of references by ~30% and reassessed the balance of new papers and old reviews

(see comment 2); perhaps this may elevate the level of sophistication of our communication towards a level befitting the reviewer's expectations.

1)The authors talk about various mechanisms of heterocellular coupling including connexins, ephaptic signaling, nanotubes etc but a discussion should be made of how these would electrically differ in vitro and most importantly in vivo in terms of functional outcomes. I understand that modeling data may not be able for all of them but still a discussion should be made.

We believe there may have been a misunderstanding regarding the distinction between morphological substrates and electrical effects of heterocellular coupling. We address substrates (e.g. lines 242 to 252), and key NM (lines 403 to 423) or CM properties (lines 436 to 454) that determine heterocellular electrical interaction effects.

2) A lot of the background on experimental observations can be shortened. It is just a rehash of old papers and no insights are obtained.

We respectfully disagree with the reviewer's suggestion. Modelling work must be well-informed by experimental insight, and we believe that presenting the latter provides essential context for the former. Discussing modelling in isolation from experimental data (and their limitations) seems futile to us. Moreover, we note that many of the reviewer's comments, below, focus on experimental aspects. We take this as indication of the need to sharpen, rather than reduce, this part of the review to ensure clarity.

We do not share the view that our citations underlying experimental insight are disproportionately old: the median publication year of the references cited in the original experimental section 2.2 was 2014 (now 2015, see Figure 1).

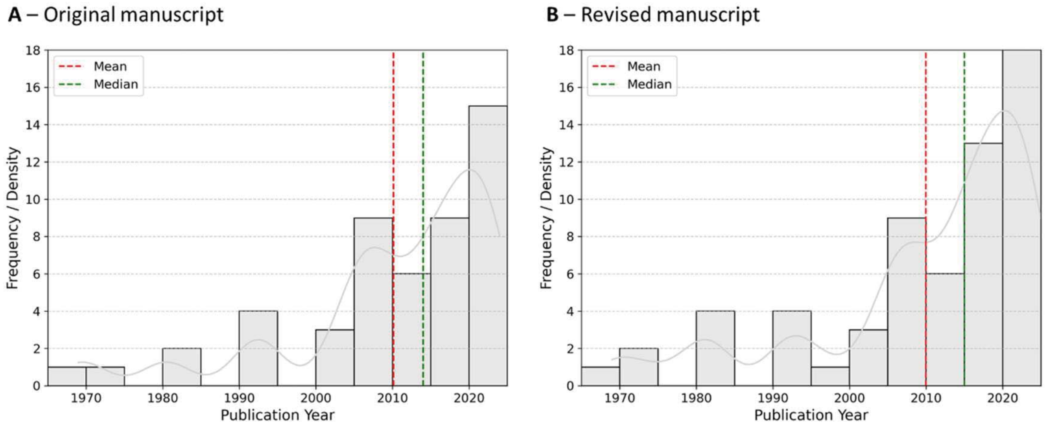

Figure 1: Distribution of publication years for references cited in Section 2.2 (Experimental Observations) in the original paper (A) and the revised version (B). The histogram shows the number of cited papers per 5-year interval. The red dashed line indicates the mean publication year (2010), and the green dashed line marks the perhaps more important median publication year (2014 for the original paper and 2015 for the revised revision).

3)The discussion about Connexins/Cx43 are confusing. Cx43 couples fibroblasts and myocytes and this could be arrhythmogenic but increased Cx43 could have beneficial effects on the scar tissue in terms of myocardial conduction velocity. These sections should be explained

We thank the reviewer for pointing out that one should not take an understanding of divergent effects of heterocellular Cx-coupling in different patho-physiological settings for granted. We have added dedicated text (lines 318 to 325 and 631 to 641) to clarify that, depending on the context, increased heterocellular coupling – e.g. via Cx43 – may have beneficial effects in scar tissue by enhancing trans-scar conduction, while in the context of atrial ablation, reducing heterocellular coupling may be functionally advantageous.

4) I think a section should be devoted to the physiological context of heterocellular coupling

We thank the reviewer for this suggestion, which we address in lines 230 to 239. We highlight the paucity of clear-cut evidence on CM-NM electrical interaction in non-injured working myocardium, suggesting that such interactions may be unlikely to have substantial electrophysiological effects in physiological conditions. We discuss that the picture may differ in specific NM-rich sub-regions of the heart, such as the sinoatrial and atrioventricular nodes.

a) What are the triggers in vivo that could change the RMP of non-myocytes (stretch, ROS??). Does Change of RMP expected to occur slowly or rapidly in these instances and the single sided and double sided coupling model should be discussed in these contexts to reflect on myocyte depolarization?

We thank the reviewer for pointing this. In lines 700 to 723, we now discuss potential *in vivo* causes on NM RMP changes, including mechanical stimuli (*via* stretch-activated channels), paracrine signaling, and inflammatory mediators—an area that needs further experimental and computational investigation.

b) The above discussion has also implications for recent high profile papers (Wang et al. Science, 1480-87 and Hulsman et al (Cell, 510-522). How physiological is the large current being generated in vivo in non-myocytes using these optogenetic platforms?

The optogenetic tools used in these studies are used as interventions to assess the principal presence of electrotonic coupling between NM and CM *in situ*, first shown functionally by Quinn et al. Of course, the whole-cell current induced by light can be titrated by illumination intensity, such as to study physiological effects of more subtle changes in membrane potential. We extended the relevant discussion, lines 738 to 743.

c) What are boundary conditions for sustained arrhythmogenesis to occur via myocyte-non myocyte coupling? What are the kinds of arrhythmias that can be generated potentially via myocyte-non-myocyte coupling? What would be the initiating events in these instances?

We discuss a number of studies looking at mechanisms favoring arrhythmia induction and perpetuation in section 3.1 and 3.2, but we don't think it is possible to boil them down into a succinct set of triggers, sustaining mechanisms, or boundary conditions beyond what is established in the general canon of arrhythmogenesis. This is an area where more targeted research is needed.

d) Ephaptic coupling in disease conditions is thought to have more relevance in cardiac pathophysiology, perhaps the role of myocyte-non-myocyte and ephaptic coupling in healthy hearts versus diseased hearts should be discussed.

The Special Issue of JP contains a paper dedicated to ephaptic coupling; we are now referring to this manuscript by acknowledged experts in this field. At the same time, we have elaborated a bit more on ephaptics where effects seem to be of particular relevance in the context of our communication (e.g. lines 247 to 282).

e) Coupling properties with the cell type: myofibroblasts versus fibroblasts versus macrophage (how would the coupling outcomes differ)

The coupling effects depend on key electrophysiological properties – both of NM and CM. For NM, the intrinsic resting membrane potential, membrane capacitance, and the strength of coupling to CM chiefly determine electrical interaction effects. Where these differ systematically between NM subtypes, effects of electrical interaction would differ correspondingly. A discussion of main determinants of electrophysiological interaction effects is provided in lines 402 to 526.

5) Fig 2 should be removed. It serves no purpose rather than advertising some papers. Better to put in some conceptual figures for the reader on myocyte-non myocyte coupling discussing any of the physiological issues discussed above or in addition.

We take the reviewer's comment as a prompt to try and improve Fig.2 information content. We are offering more context in the legend, and modified the figure to include the first model where both resistive currents and electric fields mechanisms were incorporated (Kucera *et al.*, 2002) and a recent publication that develops 3D finite element models incorporating nanoscale structures of CM-CM ephaptic coupling (Ivanovic & Kucera, 2021).

6) Transcar conduction is an interesting concept and may be clinically very important and this should be discussed in context of single sided and double sided coupling models raised by the author which I think is a good way of describing it.

We agree. In our revision, we have tried to link observations more systematically to the context of 'sidednes' of coupling throughout the text (e.g. lines 160 to 166).

7) Source sink relationships and how it affects outcomes of myocyte-non myocyte coupling should be discussed especially with regard to the diseased heart.

We agree and have expanded this in line with the reviewer's comments throughout the text (e.g. lines 596 to 598).

Referee #2:

This manuscript provides a valuable overview of current studies on heterocelular coupling in the heart, focusing on the electrical interactions between cardiomyocytes and non-myocytes (fibroblasts and macrophages) in both experimental and computational settings. It outlines the limitations of existing computational models and offers insightful perspectives on the future development of this field. The review effectively bridges clinical relevance, experimental evidence, and computational modelling, highlighting the importance of heterocellular coupling in cardiac electrophysiology. The following points require consideration by the authors.

We thank the reviewer for this generous assessment.

Title:

The manuscript integrates both experimental and computational studies, with each contributing nearly 50%. The current title specifically highlights the computational modelling and may not fully reflect this balance. Consider revising it to better align with the content of the review.

We agree that the original title placed disproportionate emphasis on computational modelling, whereas the manuscript integrates both experimental and computational studies. To better reflect this integration, we have revised the title by replacing "Computational Modelling" by "Models", as we agree that both experimental and computational models drive conceptual insight into cardiac NM-CM electrical interactions.

Introduction:

• Conceptual Re-assessment (Line 42): Elaborate on what the "conceptual re-assessment" entails regarding cardiac electrophysiology. Provide a clearer

statement about why the existing view needs to be revised in light of the evidence of non-myocyte effects.

We expanded this in line with the reviewer's comments (lines 48 to 50). Specifically, we now clarify that this re-assessment involves moving beyond the traditional CM-centric framework of cardiac electrophysiology to one that incorporates relevant contributions from various NM.

Clinical and Experimental Evidence of Cardiac Heterocellular Coupling:
• Clinical Evidence (Lines 71-73): Replace the reference to the review paper with citations to original research articles that provide experimental evidence of structural remodeling in cardiac diseases.

We fully appreciate the reviewer's suggestion and agree that seminal original research articles should be cited (e.g. see lines 79 to 82).

• Section Title (Lines 93-94): Since the last sentence in Lines 93-94 states that it is unknown if the clinical findings are based on heterocellular coupling, the section title may need to change as there is no direct clinical evidence.

We agree and have amended this. We renamed the section *Clinical Motivation* to emphasize that the findings discussed raise clinically relevant questions, even though causal links of heterocellular coupling and clinical findings are not general established.

Figure 1 Legend:

• Change "I" to lowercase "i" in "(green dots in I and ii)".

Many thanks – corrected as suggested.

• Justify why zero-sided coupling may dominate in healthy hearts (provide evidence or reasoning). Is there a citation?

We expanded this in line with the reviewer's comments (e.g. lines 139 to 142), and highlight the paucity of data in this context (lines 230 to 239). We have also modified legend of Figure 1 to be more precise.

• Replace the last "while" with "and".

Many thanks – corrected as suggested.

Experimental Observations

• Cx45 Expression (Line 171): Specify whether Cx45 expression increases or decreases.

We thank the reviewer for pointing this out and have clarified the text (line 191).

• *Direct evidence of coupling (Lines 188-180): "...demonstrating the presence of heterocellular coupling in the heart." Is direct electrotonic coupling shown or does the data show evidence of electrical interactions?*

Fair point. We have revised the statement accordingly (line 215 to 218).

• *KCa3.1 (Line 199): Correct "gene" to "channel protein." KCNN4 is the gene name.*

We thank the reviewer for pointing this out.

• *Capacitative Coupling (Lines 202-204): The authors should clarify their definition of capacitative coupling and how it relates to emerging concepts of ephaptic coupling in the heart. Both involve electrical field effects, ephaptic coupling, particularly in structures like the perinexal ephapse at gap junction edges, encompasses electrophysiological interactions that resemble capacitative coupling in some respects. Could the concepts introduced here as independent be the same thing? An acknowledgment of this unifying possibility as embodied by the perinexus would be useful.*

We expanded this in line with the reviewer's comments (e.g. lines 240 to 246), where we explain the main differences between these two mechanisms.

• *Relevance of Capacitative Coupling (Line 205): Explain briefly why capacitative coupling was deemed unlikely to be of pathophysiological relevance (cite the study's reasoning).*

We expanded this in line with the reviewer's comments (lines 247 to 252). We explain that model calculations showed that current generated by capacitive coupling was significantly smaller than that through a single gap junctional channel.

• *Ephaptic Coupling (Lines 209-212): Acknowledge the uncertainty surrounding ephaptic coupling between different cell types. Rephrase the statement to treat the existence of heterocellular ephaptic coupling as a research question.*

• *Punctate Connections: Clarify what "punctate connections" refers to. Does it refer to gap junction plaques or other types of connections?*

We expanded these two points in line with the reviewer's comment lines (253 to 271). Specifically, we discuss the existence of heterocellular ephaptic coupling as a potential mechanism, highlighting that its formation would be constrained by the presence of a basement membrane covering the CM surface both *in vivo* and *in vitro*. For ephaptic interactions to occur between CM and NM, this barrier would need to be ruptured, enabling sufficiently close membrane approximations to

support formation of diffusion-limited extracellular fluid volumes. Additionally, we clarified the term "punctate connections" to refer to discrete, localized sites of close membrane contact between CM and NM, as reported in the literature.

• *Co-cultures: In Line 227, please use co-cultures instead of cultures.*

Many thanks – corrected as suggested.

• *Line 266 "Asses" - :) maybe assess is the word you should settle on ?*

Oops – corrected as suggested.

• *Resting and Action Potential Properties*

Subtitle: Consider changing the subtitle to something like "Electrical Properties of Heterocellular Coupling" to better reflect the section's content. Action potential associated with heterocellular coupling is reflected by dynamics of ionic current.

We thank the reviewer for this helpful suggestion.

• *Equation (Line 292): Clarify if the equation given is different from the one in the appendix of the cited paper (Kohl et al., 1994) or provide the correct citation.*

We aim to provide a general equation to model heterocellular coupling. This formulation has been widely used in the literature, and several papers could be cited to support it. We have revised the text accordingly (lines 379 to 381) to clarify this point.

• *NM vs. Fibroblasts: As suggested, use "fibroblasts" instead of "NM" when discussing computational models involving fibroblasts for clarity. When the NM-CM computational model was discussed, the NM seem to refer to fibroblasts. By contrast, MΦ was used when a MΦ-CM computational model is discussed. I would suggest using fibroblasts instead of NM in the computational models involving fibroblasts.*

We have reviewed the use of general and specific NM designations throughout the manuscript to be more consistent - many thanks.

Cardiac Conduction

• *Experimental Findings: Suggest moving the first paragraph in this section, which discusses experimental effects of NM on cardiac conduction, to the section on Experimental Observations for better organization. The first paragraph in this section basically discussed effects of NMs on cardiac conduction in experimental conditions. Since this section primarily focuses on computational models, this paragraph may be more suitable to be placed somewhere in the section of Experimental observations.*

Thank you for the suggestion. We have restructured the section accordingly and relocated the paragraph discussing the experimental effects of NM on cardiac conduction to Section 2.2 (Experimental Observations), as the reviewer recommended. We agree that this change improves overall organization and clarity of the manuscript.

• *"Quantitatively" (Lines 265 and 444): Consider replacing the term "quantitatively" to better emphasize the significance of computational models compared with experimental models. For example, a computational model can be used to deeply investigate the complex mechanisms that the experimental model cannot achieve - "quantitatively" may not fully convey this important nuance.*

We agree that this term may not fully reflect the strength of computational models and we have revised the wording to better emphasize the depth and mechanistic insight that computational models can offer (lines 551 to 553).

Figure 2:

• *Improve the image resolution.*

We have re-exported Figure 2 at higher resolution and will double-check the final submission format to avoid compression artifacts.

• *Ensure the arrows in the circuit diagram for the active model are bi-directional to indicate current flow through gap junctions.*

Corrected - many thanks.

• *Clarify whether the modeling circuits are also suitable for MΦ-CM interaction. If not, consider adding a circuit specifically for MΦ-CM interaction.*

The modeling circuits presented are intended to provide a general conceptual framework illustrating that NM, such as macrophages or fibroblasts, express distinct ion channel populations. We have clarified this in the figure legend.

• *The middle panel in Figure 2: It should be Hulsmans, not Hulsman. Also, I don't think Hulsmans, et al. provided a 3D computational model in that paper. Please clarify if I am wrong.*

Corrected – many thanks.

• *The authors provide thoughtful perspectives regarding future directions for computational modeling of heterocellular coupling. As mentioned earlier, existence of ephaptic coupling between myocytes and nonmyocytes remain unclear, the future computational modelling may thus also consider incorporating*

ephaptic coupling. There are useful recent mathematical models incorporating nanoscale structures of myocyte-myocyte ephaptic coupling that could also be of service in translation to non-canonical heterocellular contacts (PMID: 34533834, PMID: 34264306).

We agree that this is an important and still insufficiently understood aspect of cardiac electrophysiology. We have added a brief discussion of this in the revised manuscript (lines 253 to 271 and 500 to 508) and included relevant papers in Figure 2.

• Authors may also consider to consistently use the first or last author for the references to avoid confusion. For example, I could not find Trayanova et al, 2011 in the citations. Maybe this citation is to the paper of McDowell, et al, 2011 in Line 811.

Absolutely – sorry for the confusion; corrected.

Present Limitations and Future Directions

• Computational Efficiency: While the review mentions the need for computationally efficient multiscale models, it could elaborate on specific techniques or approaches that could be used to achieve this goal (e.g., model order reduction, parallel computing, etc.).

Thank you for the suggestion. We have extended section 4 (lines 669 to 673) accordingly.

• Ephaptic Coupling: As suggested, future computational modelling may consider incorporating ephaptic coupling.

We have addressed ephaptic coupling at various points throughout the manuscript. Additionally, we reference another review within the special issue that focuses specifically on this topic, providing a broader context for future computational modeling efforts and include a discussion in section 4 (lines 688 to 690).

Additional Points for Consideration

• Gap Junction Conductance Variability: The review mentions that the precise in vivo value for heterocellular coupling is unknown. Interesting there is equal uncertainty on measurements of GJ conductance levels between myocytes. This was discussed in a recent paper from Poelzing's group (PMID: 37681314), which when surveying historical reports concluded that conductance levels used in many models may be over-estimated by as much as an order of magnitude. Discuss the implications of this uncertainty for model predictions and suggest ways to address this issue (e.g., sensitivity analysis, uncertainty quantification).

We expanded this in line with the reviewer's comments (lines 679 to 687).

• *Mechano-Electrical Feedback: The review could also discuss the potential role of mechano-electrical feedback in heterocellular coupling. How do mechanical forces affect the electrical properties of cardiomyocytes and non-myocytes, and how can this be modeled?*

We thank the reviewer for the comment. Indeed, the role of mechano-electrical coupling in heterocellular coupling is an important topic that was not explicitly addressed in our original manuscript. We have now included a discussion (lines 436 to 454 and 700 to 723). We also highlight recent modelling approaches that integrate tissue mechanics with electrical dynamics, and we emphasize the need for future studies to better characterize mechanosensitive properties of NM.

END OF COMMENTS

Dear Peter,

Re: JP-TR-2025-287295R1 "Computational Modelling of Cardiomyocyte-Nonmyocyte Electrical Interactions" by Ana Simón-Chica, Axel Loewe, and Peter Kohl

We are pleased to tell you that your paper has been accepted for publication in The Journal of Physiology.

IMPORTANT

*** We seem to be missing the author profile to accompany the author photograph. Please can you send this over? It doesn't need to be long (100 words). Can you please email to Diana at the office? (jp@physoc.org) - thank you!

TRANSPARENT PEER REVIEW POLICY: To improve the transparency of its peer review process, The Journal of Physiology publishes online as supporting information the peer review history of all articles accepted for publication. Readers will have access to decision letters, including Editors' comments and referee reports, for each version of the manuscript, as well as any author responses to peer review comments. Referees can decide whether or not they wish to be named on the peer review history document.

The last Word (or similar) version of the manuscript provided will be used by the Production Editor to prepare your proof. When this is ready you will receive an email containing a link to Wiley's Online Proofing System. The proof should be thoroughly checked and corrected as promptly as possible.

Authors should note that it is too late at this point to offer corrections prior to proofing. Major corrections at proof stage, such as changes to figures, will be referred to the Editors for approval before they can be incorporated. Only minor changes, such as to style and consistency, should be made at proof stage. Changes that need to be made after proof stage will usually require a formal correction notice.

All queries at proof stage should be sent to: TJP@wiley.com.

Are you on Twitter? Once your paper is online, why not share your achievement with your followers? Please tag The Journal (@jphysiol) in any tweets and we will share your accepted paper with our 30,000 followers!

Yours sincerely,

Bjorn Knollmann
Senior Editor
The Journal of Physiology

P.S. - You can help your research get the attention it deserves! Check out Wiley's free Promotion Guide for best-practice recommendations for promoting your work at www.wileyauthors.com/eeo/guide. You can learn more about Wiley Editing Services which offers professional video, design, and writing services to create shareable video abstracts, infographics, conference posters, lay summaries, and research news stories for your research at www.wileyauthors.com/eeo/promotion.

IMPORTANT NOTICE ABOUT OPEN ACCESS: To assist authors whose funding agencies mandate public access to published research findings sooner than 12 months after publication, The Journal of Physiology allows authors to pay an Open Access (OA) fee to have their papers made freely available immediately on publication.

The Corresponding Author will receive an email from Wiley with details on how to register or log-in to Wiley Authors Services where you will be able to place an order.

You can check if your funder or institution has a Wiley Open Access Account here: https://authorservices.wiley.com/author-resources/Journal-Authors/licensing-and-open-access/open-access/author-compliance-tool.html.

EDITOR COMMENTS

Reviewing Editor:

The authors have adequately addressed the reviewers' concerns, resulting in a improved manuscript, that represents a timely and potentially impactful review from leaders in the field.

Senior Editor:

The revised manuscript is now acceptable. Thank you for your excellent contribution to the Journal!

REFEREE COMMENTS

Referee #1:

The authors have taken the effort to address most of my comments and the review is much better.

Referee #2:

The authors have satisfactorily addressed reviewer points, providing detailed, transparent, and specific responses. Where the authors disagreed with review points, they explained their reasoning. The revised manuscript reflects these changes and is improved in clarity, scope, and technical detail.
